# Escaping Death: How Cancer Cells and Infected Cells Resist Cell-Mediated Cytotoxicity

**DOI:** 10.3389/fimmu.2022.867098

**Published:** 2022-03-23

**Authors:** Karoliina Tuomela, Ashley R. Ambrose, Daniel M. Davis

**Affiliations:** The Lydia Becker Institute of Immunology and Inflammation, The University of Manchester, Manchester, United Kingdom

**Keywords:** cell-mediated cytotoxicity, cytolytic T cells, natural killer cells, lymphocytes, cancer, resistance, viral infection, immune synapse

## Abstract

Cytotoxic lymphocytes are critical in our immune defence against cancer and infection. Cytotoxic T lymphocytes and Natural Killer cells can directly lyse malignant or infected cells in at least two ways: granule-mediated cytotoxicity, involving perforin and granzyme B, or death receptor-mediated cytotoxicity, involving the death receptor ligands, tumour necrosis factor-related apoptosis-inducing ligand (TRAIL) and Fas ligand (FasL). In either case, a multi-step pathway is triggered to facilitate lysis, relying on active pro-death processes and signalling within the target cell. Because of this reliance on an active response from the target cell, each mechanism of cell-mediated killing can be manipulated by malignant and infected cells to evade cytolytic death. Here, we review the mechanisms of cell-mediated cytotoxicity and examine how cells may evade these cytolytic processes. This includes resistance to perforin through degradation or reduced pore formation, resistance to granzyme B through inhibition or autophagy, and resistance to death receptors through inhibition of downstream signalling or changes in protein expression. We also consider the importance of tumour necrosis factor (TNF)-induced cytotoxicity and resistance mechanisms against this pathway. Altogether, it is clear that target cells are not passive bystanders to cell-mediated cytotoxicity and resistance mechanisms can significantly constrain immune cell-mediated killing. Understanding these processes of immune evasion may lead to novel ideas for medical intervention.

## Introduction

Cytotoxic lymphocytes, including cytotoxic T lymphocytes (CTLs) and natural killer (NK) cells, are able to directly lyse malignant or infected cells using multiple mechanisms. Granule-mediated cytotoxicity involves the release of lytic granules containing perforin and granzymes, while death receptor-mediated cytotoxicity utilises tumour necrosis factor-related apoptosis-inducing ligand (TRAIL) or Fas ligand (FasL) that bind death receptors on the surface of the target cell (1–3). In addition to these classic cytotoxic pathways, there is increasing evidence that the tumour necrosis factor (TNF) pathway also significantly contributes to lymphocyte cytotoxicity (4). Activation of these pathways can lead to cell death in several forms, including necrosis, apoptosis, necroptosis, and pyroptosis (5).

Lymphocyte cytotoxicity is triggered upon contact with a cancerous or infected target cell if sufficient activating signals are received. For CTLs, this requires initial priming by antigen presenting cells followed by T cell receptor recognition of specific target cell antigens, whereas activating receptors on NK cells recognise a range of germline-encoded ligands without prior activation (6, 7). The area of cell-cell contact between a lymphocyte and a target cell is termed an immune synapse, on account of it being a highly organised interface involving cytoskeletal and membrane rearrangement (8–12). Integration of multiple activating and inhibitory pathways within the lymphocyte determines the outcome of this interaction with a target cell (13, 14). Malignant or infected cells may, therefore, evade immune recognition through the downregulation of activating signals or the upregulation of inhibitory signals. These immune evasion mechanisms – for example reducing antigen recognition, triggering immune checkpoints, secreting immunosuppressive cytokines, as well as excluding immune cells from the microenvironment – have been extensively reviewed elsewhere (15–18). And of course, many immunotherapies have been developed to target these types of immune escape mechanisms, such as checkpoint inhibitors, adoptive cell therapy, and cancer vaccines, all of which aim to enhance immune cell activation (6, 19).

However, evasion of lymphocyte cytotoxicity may also occur downstream of immune cell activation. Even if a cytolytic response is triggered by the lymphocyte, target cells are not passive bystanders to lymphocyte cytotoxicity. This is because mechanisms of cell death generally rely on active pro-death signalling within the target cell (5). Indeed, evasion of cell death and apoptosis is considered a critical hallmark of cancer (20). Therefore, resistance to lymphocyte attack may arise through resistance to the mediators of cytotoxicity, including perforin, granzymes, and death receptor ligands. Here we review the molecular details behind cell-mediated killing and then examine our current understanding of how target cells may resist these cytotoxic processes as immune evasion strategies.

## Mechanisms of Lymphocyte Cytotoxicity

### Granule-Mediated Cytotoxicity

**Figure 1:**
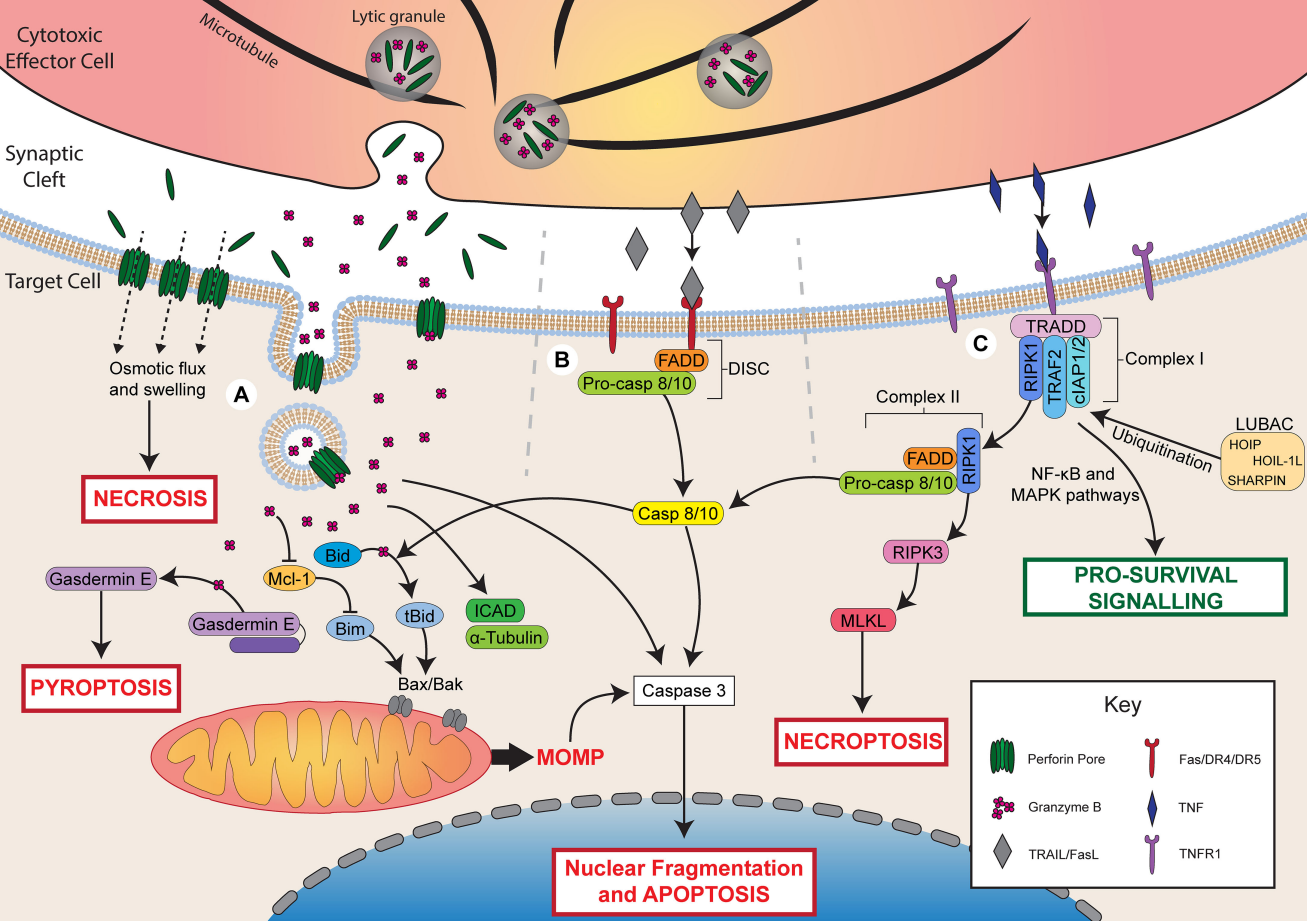
Mechanisms of lymphocyte cytotoxicity. Following activation, cytotoxic effector cells can kill through granule-mediated cytotoxicity, death receptor-mediated cytotoxicity, or TNF-mediated cytotoxicity. **(A)** During granule-mediated cytotoxicity, perforin and granzymes are released from lytic granules into the synaptic cleft. Perforin forms pores in the target cell membrane. At high concentrations of perforin, osmotic flux through pores leads to cell swelling and necrotic cell death. Perforin can facilitate the uptake of granzyme B through direct diffusion or endocytosis. Granzyme B directly cleaves caspase 3 to induce apoptosis or triggers the mitochondrial apoptotic pathway *via* Bid cleavage into tBid. tBid recruits Bax/Bak leading to mitochondrial outer membrane permeabilization (MOMP) and apoptosis. Granzyme B may also degrade Mcl-1 releasing Bim to activate MOMP. Granzyme B can also cleave ICAD contributing to DNA damage, α-tubulin leading to cytoskeletal degradation, or gasdermin E, which forms pores in the cell membrane to induce pyroptosis. **(B)** Ligation of death receptors (Fas/DR4/DR5) by FasL or TRAIL triggers assembly of the death-inducing signalling complex (DISC) composed of FADD and pro-caspase 8/10. Caspase 8/10 induces apoptosis *via* direct caspase 3 cleavage or the mitochondrial apoptotic pathway *via* Bid cleavage. **(C)** Ligation of TNFR1 by TNF triggers the assembly of complex I (TRADD, RIPK1, TRAF2, cIAP1/2). LUBAC ubiquitinates complex I components leading to pro-survival signalling *via* NF-κB and MAPK pathways. In the absence of ubiquitination, RIPK1 dissociates and forms complex II with FADD and pro-caspase 8/10. Cleavage of pro-caspase 8/10 triggers apoptosis by the same pathways as FasL/TRAIL. In the presence of insufficient pro-caspase 8, RIPK1 can also recruit RIPK3, which activates MLKL to trigger necroptosis.

Following activation, effector cells polarise their microtubule organising centre (MTOC) and lytic granules towards the immune synapse, then release the contents of these granules across the synaptic cleft (21–23). Granule-mediated cytotoxicity is dependent upon the release of perforin and granzymes from granules contained within cytotoxic lymphocytes (**Figure 1**). Perforin is a pore-forming protein that forms ring-shaped lesions capable of mediating ion flux as well as the uptake of larger molecules, such as granzymes (24). Granzymes are a family of serine proteases that cleave a variety of target proteins within cells in order to induce apoptosis. Five granzymes have been identified in humans, A, B, H, K, and M, but granzyme A and B have been characterised most extensively (25, 26).

Granule-mediated cytotoxicity can result in cell death through two mechanisms (**Figure 1**). The first is necrotic cell death induced by rapid osmotic flux through perforin pores and membrane rupture, which can be observed upon exposure to high concentrations of perforin (27, 28). The second is apoptotic cell death induced by perforin-mediated uptake of granzyme B into the target cell. Two primary models have been proposed to account for the entry of granzyme B into target cells: direct diffusion through perforin pores in the cell membrane, or perforin-induced endosomal uptake of granzymes. Perforin pores observed by electron microscopy have been measured to be physically large enough to permit the diffusion of granzymes into the target cell cytoplasm (29). Furthermore, intracellular granzyme B activity can be observed within minutes of adding exogenous granzyme B and perforin to target cells (30), which is faster than has been observed for endosomal uptake of granzyme B (31). Conversely, perforin has been shown to trigger a calcium-dependent membrane repair response that triggers the endocytosis of granzyme B, and granzyme B-positive endosomes can be observed within target cells following interaction with NK cells (31–33). It is possible that these two pathways work in parallel or in different cellular contexts to facilitate the uptake of granzyme B into target cells. Recent evidence demonstrated that perforin and granzyme can also be secreted from CTLs and NK cells in complexes, termed supramolecular attack particles (SMAPs), bound by the adhesive glycoprotein thrombospondin-1 (34, 35). Further understanding of SMAP biology may shed light on whether or not the two models of granzyme B delivery work synergistically or independently.

Once within a target cell, granzymes can trigger cell death through several pathways, and the specific pathways that are activated are dependent on the identity of the granzyme. Granzyme B is the most potent member of the granzyme family and can induce apoptosis within minutes of delivery (30). This occurs through either direct cleavage of caspases by granzyme B or *via* activation of the mitochondrial pathway of apoptosis. Direct cleavage of caspases, such as caspase 3, one of the executioner caspases, is the primary mechanism by which mouse granzyme B induces apoptosis (25). Caspase 3 cleaves several targets including inhibitor of caspase-activated DNase (ICAD) and gelsolin, which leads to DNA damage and cytoskeletal disruption, respectively (36). Both human and mouse granzyme B can also trigger the mitochondrial apoptotic pathway, which is characterised by mitochondrial outer membrane permeabilization (MOMP) (37–39).

MOMP is regulated by the Bcl-2 family, which is made up of three classes: pro-apoptotic BH3 proteins (e.g. Bid, Bim), pro-apoptotic effector proteins (e.g. Bax, Bak), and anti-apoptotic proteins (e.g. Bcl-2) (40). Granzyme B triggers MOMP by cleaving Bid into its active, truncated form (tBid) (37). After cleavage, tBid recruits the pore-forming effector proteins, Bax and Bak, to the mitochondrial membrane where they form pores that mediate MOMP and the release of additional apoptotic mediators, such as cytochrome c (40). This process can be opposed by the action of anti-apoptotic Bcl-2 proteins, which bind and inhibit pro-apoptotic Bcl-2 members (40). Importantly, granzyme B can also induce degradation of these anti-apoptotic Bcl-2 proteins, such as Mcl-1, leading to the release of the pro-apoptotic BH3 protein, Bim, which activates Bax/Bak and triggers apoptosis (38, 41). A granzyme B-mediated mitochondrial apoptotic pathway, independent of Bax/Bak, has also been identified, and occurs through cleavage of mitochondrial proteins involved in the electron transport chain and production of reactive oxygen species (42–44).

Granzyme B can also cleave several additional targets that contribute to cell death. For example, granzyme B can directly cleave the caspase 3 substrate, ICAD, leading to DNA damage (45) or cleave α-tubulin, causing cytoskeletal disruption during apoptosis (46, 47). Recently, granzyme B was also found to cleave and activate the pore-forming protein gasdermin E, leading to an alternate form of cell death, pyroptosis, through the formation of gasdermin pores in the cell membrane (48, 49). Pyroptosis is a more inflammatory form of cell death compared to apoptosis and relies on the formation of pores in the cell membrane by members of the gasdermin family (5, 50). Cleavage of gasdermin E by granzyme B is a potent mechanism by which cytotoxic lymphocytes can kill cancer cells and control tumour growth (48).

Compared to granzyme B, granzyme A is a far less efficient inducer of cell death and triggers apoptosis at a slower rate (51). Granzyme A-induced apoptosis is caspase-independent and, although its targets are not fully defined, is mediated by cleavage of a variety of nuclear, mitochondrial, and cytosolic proteins (52, 53). Recently, granzyme A was also shown to trigger pyroptosis of cancer cells through cleavage of the pore-forming protein, gasdermin B (54, 55). In murine tumours, gasdermin B expression synergised with checkpoint blockade to promote tumour clearance by cytotoxic lymphocytes (54). Likewise, in the context of infection by *Shigella flexneri*, granzyme A secreted by NK cells was found to cleave gasdermin B within the infected cell (55). Cleaved gasdermin B demonstrated microbiocidal activity by forming pores within the bacterial membrane in order to protect the host cell. Less is known about the function and role of the other granzymes (K, H, and M) expressed by human lymphocytes.

### Death Receptor-Mediated Cytotoxicity

Cytotoxic lymphocytes may also kill target cells through the expression of ligands for death receptors. Two prototypical ligands have been identified that mediate apoptosis: FasL, which binds the Fas receptor, and TRAIL, which binds death receptors 4 and 5 (DR4/5) (56). Although FasL and TRAIL bind different receptors, both ligands trigger similar pro-apoptotic signalling (**Figure 1**). Both FasL and TRAIL are transmembrane proteins that belong to the TNF superfamily and can be expressed on cytotoxic immune cells upon cytokine stimulation or interaction with a target cell (1, 57). Upon binding of FasL or TRAIL to their respective receptors, assembly of the death-inducing signalling complex (DISC) is triggered. The DISC consists of the death receptor, Fas-associated death domain protein (FADD), and pro-caspase 8 or 10 (56). The DISC mediates cleavage of pro-caspase 8/10 to release the active caspase, which can then activate the executioner caspases 3, 6, and 7. Caspase 8 can further amplify apoptotic signalling by cleaving Bid to activate the mitochondrial pathway of apoptosis, similar to granzyme B (56).

### TNF-Mediated Cytotoxicity

TNF is a cytokine capable of inducing both pro-survival and pro-death signalling depending on the precise cellular context. Although the receptors for TNF, TNF-R1 and TNF-R2, belong to the same family as the receptors for FasL and TRAIL, the downstream signalling pathways are distinct (4). Of the two receptors for TNF, only TNF-R1 is able to trigger cell death through its cytoplasmic death domain, which recruits a key adaptor protein, TNF receptor-associated death domain (TRADD) (4). Conversely, both TNF-R1 and TNF-R2 contain a TNFR-associated factor (TRAF) binding site that recruits TRAF1/2, which is involved in triggering pro-survival signalling *via* the NF-κB and MAPK pathways.

The pro-survival and pro-death signalling pathways controlled by TNF-R1 ligation are mediated by the assembly of two signalling complexes, complex I and II, respectively (4, 58). Complex I, which mediates pro-survival signalling, is composed of several proteins, including receptor interacting serine/threonine protein kinase 1 (RIPK1), TRAF2/5, cellular inhibitor of apoptosis 1/2 (cIAP1/2), and linear Ub chain assembly complex (LUBAC). LUBAC-mediated ubiquitination of complex I components leads to the recruitment of additional kinase complexes involved in NF-κB and MAPK survival signalling (4). Survival signalling through complex I is generally the default pathway triggered by TNF-R1 ligation. However, in certain cell states, TNF-R1 signalling can switch instead to pro-death signalling mediated by complex II, which is composed of RIPK1, FADD, and pro-caspase 8 (4, 58, 59). Assembly of complex II occurs when RIPK1 is not ubiquitinated, such as in the absence of the complex I-associated proteins cIAP1/2 and LUBAC (59, 60). Non-ubiquitinated RIPK1 dissociates from TRADD and recruits FADD and pro-caspase 8 leading to similar pro-death signalling as TRAIL/FasL (4). When caspase 8 activation is not sufficient, complex II can also lead to necroptotic cell death. This occurs through autophosphorylation of RIPK1, leading to the recruitment and autophosphorylation of RIPK3 followed by activation of mixed lineage kinase domain-like (MLKL), which induces necroptosis (4). Altogether, much remains to be understood about the signalling that regulates the varying effects of TNF and how this can change in different cell states.

## Mechanisms of Resistance to Cytotoxicity

Overall, there is a reliance on active processes and signalling pathways within the target cell to execute CTL and NK cell killing. This implies that each distinct mechanism of cell-mediated killing can be open to an evasion strategy by the target cell. Indeed, malignant and infected cells develop a variety of mechanisms to evade cytolytic death.

The existence of these resistance mechanisms is readily observed when tracking interactions between cytotoxic lymphocytes and target cells *in vitro*. Even when CTLs are activated during an interaction with a target cell – as indicated by a rapid increase in calcium concentration within the effector cell – the target cell does not always die (61, 62). In some cases, CTLs may produce a sublethal hit, which is characterised by a transient calcium flux, indicative of perforin pore formation, but no cell death (62). Alternatively, in some effector-target interactions, no calcium flux could be observed in the target cell despite apparent activation of the effector cell, indicating that perforin did not form pores in the target cell membrane. A similar study demonstrated that cancer cells often recover even after a cytotoxic hit that triggers a large calcium flux, structural perturbations, and DNA damage (61). The target cancer cells were observed to rapidly restore calcium homeostasis, recover nuclear integrity after structural damage, and even repair DNA to stop apoptosis at its later stages (61). Clearly, not every interaction between a cytotoxic lymphocyte and its target results in death. While some of this survival could be attributed to stochastic variability, specific methods by which target cells can evade cytotoxicity can have a profound impact on the ability of cytotoxic lymphocytes to eliminate cancer cells and infected cells *in vitro*.

### Resisting Perforin Pore-Formation

**Figure 2:**
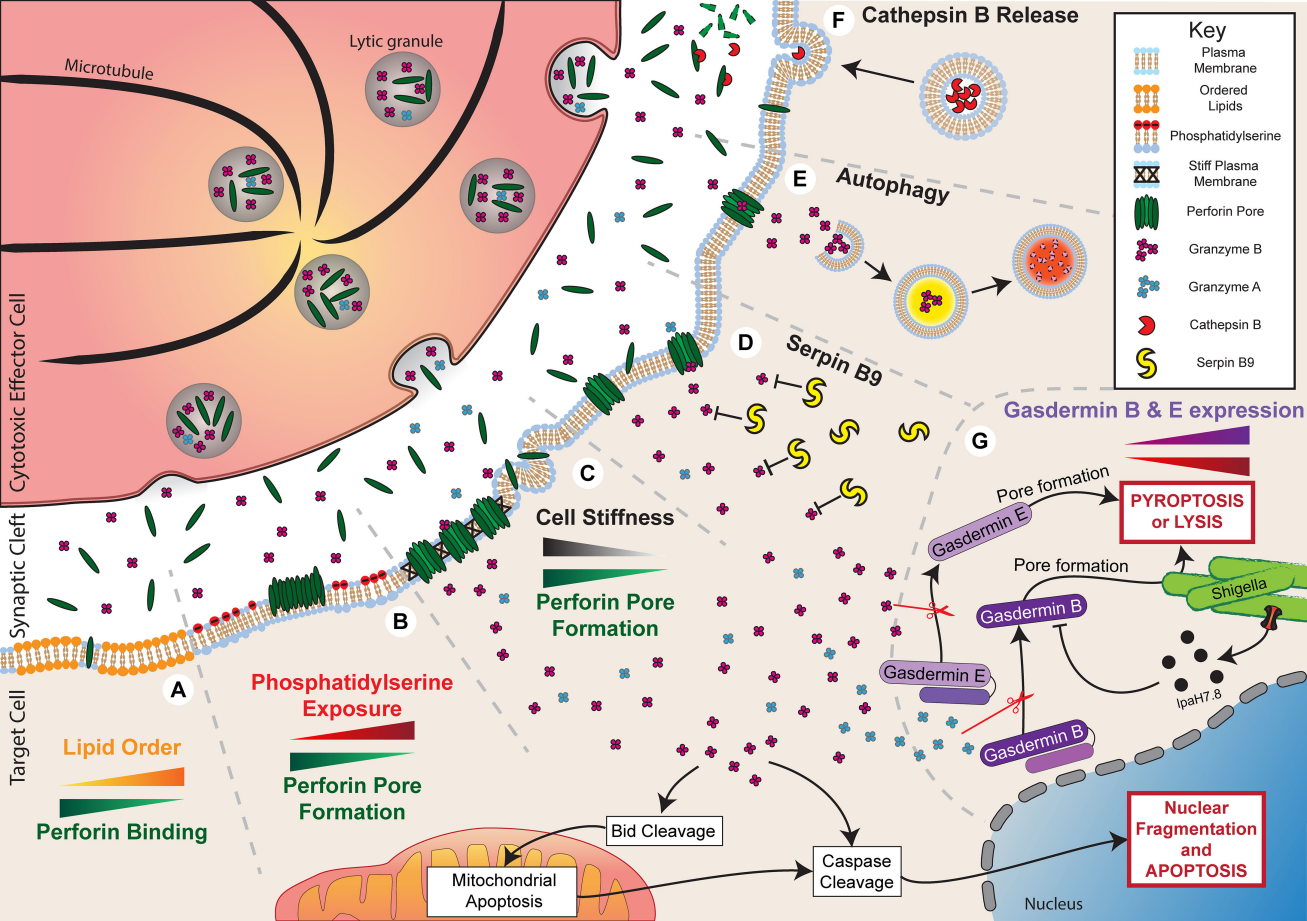
Resistance to granule-mediated cytotoxicity. Activation of a cytotoxic effector cell at an immune synapse with a target cell leads to the polarisation and secretion of lytic granules containing perforin and granzyme B. Under normal circumstances, in the absence of any resistance mechanisms, the secreted perforin will form pores in the target cell membrane and allow entry of granzyme B. This process will initiate cell death through both direct cell lysis and the activation of apoptotic pathways. Target cells can employ multiple mechanisms to evade cytotoxicity. **(A)** Increased plasma membrane lipid order to reduce perforin binding. **(B)** Externalisation of phosphatidylserine to induce perforin aggregation rather than pore formation. **(C)** Reduced cell stiffness to prevent efficient perforin pore formation. **(D)** Expression of Serpin B9 to directly inhibit Granzyme B activity. **(E)** Autophagy of Granzyme B to prevent activation of apoptotic pathways. **(F)** Secretion of Cathepsin B to degrade perforin. **(G)** Reduced gasdermin B and E expression or IpaH7.8-mediated ubiquitination and degradation of gasdermin B can reduce pyroptosis or lysis of shigella, respectively.

The formation of perforin pores in target cell membranes is the first step of granule-mediated cytotoxicity and is required for the induction of both necrosis caused by osmotic flux and apoptosis caused by granzyme B uptake. Therefore, resistance to this initial step of granule-mediated cytotoxicity has the potential to significantly reduce lymphocyte cytotoxicity. Perforin resistance was initially identified as a characteristic of cytotoxic lymphocytes, including CTLs and NK cells, which are less easily killed by purified perforin compared to various non-cytotoxic cell lines (63–65). This resistance is thought to be integral to the survival of lymphocytes when releasing their cytotoxic cargo. Resistance to perforin has also been observed in malignant cells (66–68). In studies of patient-derived leukaemia and lymphoma samples, considerable variability was observed in the ability of perforin to bind and lyse cancer cells from different patients (66, 67). Importantly, the susceptibility of cancer cells to perforin-induced lysis closely correlated with the amount of perforin bound (66), indicating that cancer cells may evade perforin by reducing binding. More recently, our own research has found that *in vitro* irradiation of cancer cells transiently reduces susceptibility to lysis by NK cells and CAR T cells by inducing resistance to perforin, possibly by preventing pore formation (68). Our current understanding of the mechanisms that mediate perforin resistance in malignant or infected cells is predominantly derived from protective mechanisms employed by cytotoxic lymphocytes. These mechanisms of perforin resistance include altered lipid order, phosphatidylserine exposure, modulation of cell stiffness, and cathepsin B-mediated degradation (**Figure 2**), each of which we will now explore in detail.

#### Lipid Order

The lipid order of a membrane is a characteristic determined by several properties: lipid packing, the rotational freedom of lipids, and the thickness of the bilayer (69). Therefore, changes to membrane composition can alter lipid order, such as increasing lipid order upon incorporation of cholesterol (70). From the time of its discovery, perforin has been known to preferentially bind to synthetic liposomes or planar lipid bilayers composed of low-order, fluid-phase lipids, such as 1,2-dioleoyl-sn-glycero-3-phosphocholine (DOPC), compared to high-order, gel-phase lipids, such as dipalmitoylphosphatidylcholine (DPPC) (71–74). This specificity for lipid order appears to play a critical role in protecting cytotoxic lymphocytes against self-harm by perforin since CTLs have particularly tightly packed and ordered membranes (72, 73). Lipid order has also been observed to particularly increase at the site of the immune synapse compared to more distal areas of the lymphocyte membrane (75–77). Replacing cholesterol in CTL membranes with a disorder-prone cholesterol variant increases perforin binding and sensitises cells to pore-induced lysis, consistent with tight lipid packing being protective against perforin pore formation (73).

Alterations in lipid packing may directly affect the susceptibility of cancer cells to perforin-mediated attack. Lymphocyte-resistant breast cancer cells, for example, have been found to increase lipid order at the site of the immune synapse and, similar to observations with CTLs, replacing cholesterol with a disorder-prone variant sensitises cancer cells to perforin-induced lysis (73, 75). More broadly, lipid composition is often highly altered during malignancy and infection. For example, multidrug resistant cancer cells frequently exhibit increased membrane lipid order due to increased cholesterol levels (78, 79). Therefore, it is possible that lipid order-mediated resistance to perforin is a common feature of cancer. However, most observations have been made *in vitro* and further work is necessary to understand whether alterations in the lipid order of cancer cell membranes can have a significant impact on tumour control by lymphocytes *in vivo*.

#### Phosphatidylserine Externalisation

Apart from altering lipid order, membrane composition can affect perforin activity through other mechanisms. Phosphatidylserine is a negatively charged phospholipid that generally localises to the intracellular leaflet of the cell membrane but can be externalised to the outer leaflet in certain cell states (80). In particular, externalisation of phosphatidylserine on the outer leaflet is often used as a marker of cell death, but it also has a variety of non-apoptotic roles for intercellular signalling (81). Importantly, phosphatidylserine can be externalised on lymphocyte membranes at the immune synapse, where it is suggested to act as a protective mechanism against perforin pore formation (73, 74, 82). Atomic force microscopy has shown that perforin is able to bind to phosphatidylserine-containing planar lipid bilayers, but it forms protein aggregates rather than membrane-spanning pores (73, 74). In addition, perforin shows little or no lytic activity against synthetic liposomes composed of high levels of phosphatidylserine (68). Other negatively charged membrane lipids, such as 1,2-dioleoyl-sn-glycero-3-phospho-(1′-rac-glycerol) (DOPG) or cholesterol sulfate, had a similar effect in preventing perforin pore formation, suggesting that the negative charge of phosphatidylserine is critical for its inhibitory effect against perforin (74).

In addition to protecting cytotoxic lymphocytes, phosphatidylserine may also be utilised by infected or malignant cells to evade attack mediated by perforin. Phosphatidylserine exposure is a common feature of cancer cells and can be further enhanced in certain circumstances, such as following anti-cancer treatment (68, 83). Externalisation of phosphatidylserine following the treatment of cancer cells with radiotherapy or cell cycle inhibitors was found to closely correlate with resistance to perforin and lymphocyte cytotoxicity, despite normal recognition and activation by NK cells and CAR T cells (68). Treatment of cancer cells with radiotherapy or cell cycle inhibitors did not affect perforin binding or membrane repair responses, suggesting that the mechanism of resistance was impaired pore formation, similar to the observed effects of phosphatidylserine in synthetic lipid membranes (68, 73, 74). Increased surface phosphatidylserine on malaria-infected erythrocytes also correlated with reduced susceptibility to perforin and reduced lysis by γδ T cells (84). The extent to which phosphatidylserine externalisation affects the elimination of target cells *in vivo* is unclear, and hard to establish. Whether this process could be targeted therapeutically is an open question.

#### Cell Stiffness

Physical properties of cells may also influence susceptibility to perforin, including cell tension or stiffness. Cell stiffness is commonly altered during malignancy, with cancer cells being relatively soft and deformable compared to healthy cells (85). Accordingly, soft CD133+ tumour-repopulating cells were found to be resistant to perforin and take up less granzyme B following interaction with T cells (86). These cells also evaded T cell cytotoxicity *in vivo* but killing could be enhanced if cells were treated with jasplakinolide, which promotes actin polymerisation and increases cancer cell stiffness. Cell stiffness can also be artificially altered by culturing cells on stiff or soft hydrogels, which directly changes the stiffness of cells to mirror the underlying substrate. Reducing stiffness in this way by culturing on a soft substrate has been shown to reduce susceptibility to perforin and lymphocyte cytotoxicity (87). It is not entirely clear why cell stiffness affects perforin lytic ability in this way, but insertion and pore formation by hydrophobic molecules, such as perforin, is known to be more energetically favourable on stiff membranes (87, 88).

Interestingly, cytotoxic lymphocytes have been found to utilise a mechanism which may counteract the reduced activity of perforin on soft cells. By using F-actin-rich protrusions, CTLs can exert lateral force on the target cell to increase membrane tension and enhance perforin pore formation (87, 89). Lytic granule secretion from CTLs was observed at the base of these protrusions so that it was spatially localised to the areas of force exertion on the target cell membrane (89). It is interesting to note that the stiffness of a target cell can also modulate NK cell and CTL activation itself, with activation significantly reduced against target cells exhibiting a soft phenotype or grown on a soft substrate (90, 91). Thus, alterations in cell stiffness at the whole-cell level, such as during malignancy, or at the nanoscale, at the immune synapse, may potently manipulate the sensitivity of cancerous or infected cells to perforin.

#### Cathepsin B

The pore-forming activity of perforin may also be reduced through direct degradation. Cysteine cathepsins are lysosomal peptidases with multiple roles in regulating immune responses (92). It was initially shown that cathepsin B, which becomes expressed on the surface of lymphocytes following degranulation, can degrade perforin in order to protect against self-harm (93). Inhibition of this surface-bound cathepsin B using the inhibitor CA074 led to enhanced CTL death after degranulation. Seemingly in contrast to this, it was later shown that CTLs from cathepsin B-null mice were not more susceptible to death following interaction with a target cell compared to cells from wild type mice (94). To reconcile these observations, it is possible that there is redundancy in the system, and perhaps compensatory mechanisms are augmented in cathepsin B-null mice, such as increased lipid order or phosphatidylserine exposure, as discussed previously.

More recent evidence has also linked cathepsin B with cancer cell resistance against lymphocyte cytotoxicity. Khazen et al. (95) showed that melanoma cells that were resistant to CTL cytotoxicity bound less perforin and took up less granzyme B despite inducing similar levels of CTL degranulation. This resistance was mediated by increased exocytosis of lysosomes or late endosomes at the immune synapse, which facilitated the secretion of cathepsin B leading to perforin degradation. Interestingly, cathepsin B is known to be overexpressed in multiple cancers, where it is associated with poor survival and metastasis (96).

Altogether, resistance to perforin through physical or degradative inhibition is an emerging aspect of immune resistance. Many of the mechanisms employed by cytotoxic lymphocytes to protect against self-harm appear to also be exploited by malignant or infected cells to inhibit perforin activity and enhance survival. However, the contribution of perforin resistance to immune escape has not yet been extensively explored *in vivo*. The ability to modify cancer cells pharmacologically to increase perforin susceptibility may be a way to increase the efficacy of lymphocyte cytotoxicity.

### Resisting Granzyme-Mediated Apoptosis

In addition to perforin, granzymes are a critical component of granule-mediated cytotoxicity. Following perforin pore formation, granzymes enter the target cell and cleave a variety of targets to potently induce cell death. As a result, reduced granzyme activity within target cells may critically constrain killing by lymphocytes. Granzyme activity may be reduced in two ways: through reduced granzyme uptake as a result of perforin inhibition, as described previously, or through direct inhibition of granzyme function. The effect of reduced granzyme uptake on cytotoxicity was clearly demonstrated by the finding that cancer cells, which were resistant to NK cell cytotoxicity, can undergo extensive cytoskeletal remodelling, which reduces granzyme uptake (97, 98). Although the underlying link between cytoskeletal remodelling and reduced granzyme uptake was not identified in these studies, cytoskeletal inhibitors were found to restore granzyme levels and cytotoxicity following interaction with NK cells. Granzyme-induced cell death may also be reduced by direct inhibition of granzyme activity. Of all the proteins in the granzyme family, the mechanisms that reduce granzyme B-mediated cell death have been characterised the most. Inhibitory mechanisms that act directly on granzyme B pathways to prevent cell death include inhibition by serpin B9, degradation through autophagy, and disruption of gasdermin-mediated pyroptosis (**Figure 2**).

#### Serpin B9

Serpin B9 (also known as serine proteinase inhibitor B9 or proteinase inhibitor 9) is the only endogenous granzyme B inhibitor that has been identified. It was initially discovered in cytotoxic lymphocytes where it protects against apoptosis by binding to and inhibiting granzyme B (99, 100). When unbound to substrate, serpin B9 exists in a semi-stable form, but it is cleaved upon binding to granzyme B causing a conformational change into its most stable form and leaving a non-functional covalently-bound serpin B9-granzyme B complex (101). Apart from in immune cells and at certain immune-privileged sites, such as reproductive organs and the eye, normal human tissue does not express serpin B9 (102). However, serpin B9 expression has been observed in multiple primary cancers, including lymphoma, melanoma, colon carcinoma, breast cancer, and lung cancer, in which it generally correlates with poor prognosis (102–107).

Overexpression of serpin B9 in various cancer cell lines results in resistance to killing by cytotoxic lymphocytes and, critically, is associated with resistance to immune checkpoint blockade in murine melanoma as well as against radiotherapy-induced type I interferon signalling (104, 108–112). Interestingly, the resistance of serpin B9-expressing cancer cells to cytotoxic lymphocytes is less evident at high ratios of lymphocytes to cancer cells (111). Furthermore, it has been shown through live imaging that multiple NK cell attacks successfully kill serpin B9-expressing target cells, while single hits are sufficient to kill targets which don’t express serpin B9 (113). This is consistent with serpin B9-mediated inhibition of cytotoxicity being overcome through increased granzyme B delivery *via* multiple lytic hits.

Although serpin B9 has primarily been described as an inhibitor of lymphocyte-derived granzyme B, it has also been shown to have a broader role in mediating tumour immune escape. For example, overexpression of serpin B9 can inhibit TRAIL-, FasL-, and TNF-mediated apoptosis through directly inhibiting caspase 8 and 10 (108, 114). Furthermore, serpin B9 can promote tumour survival through inhibition of cancer cell-intrinsic granzyme B, which can become expressed in various malignancies (107). Therefore, pharmacological inhibition of serpin B9 may aid the destruction of target cancer cells through multiple pathways. Recently, inhibition of serpin B9 has been shown to slow the development of melanoma and increase the lifespan of mice with breast, kidney and lung tumours (107).

#### Autophagy

Cancer cells may also evade cytotoxicity through autophagic pathways. Autophagy is a physiological process by which damaged or surplus proteins and organelles are degraded and recycled (115). It has a particularly important role in preventing cell death during cellular stress, such as nutrient starvation or hypoxia. Increased autophagy is also a common feature of tumorigenesis to protect against the harsh environment often present within tumours. This also enables cancer cells to maintain their highly proliferative and metabolically active states even when the microenvironment is not conducive to it (115).

Autophagy may also contribute to tumour growth by promoting immune evasion. Hypoxia-induced autophagy, for example, has been shown to correlate with resistance to CTL and NK cell cytotoxicity (116, 117). Similarly, induction of autophagy as a result of genetic inactivation of the von Hippel-Lindau (VHL) gene reduces killing by NK cells (118). Genome-wide CRISPR screens searching for genes that mediate resistance to CTL cytotoxicity have also identified a range of autophagy-related genes associated with cytotoxicity resistance (119, 120). Degradation of granzyme B may be one mechanism by which autophagy inhibits cytotoxicity. Breast cancer cells with autophagy processes stimulated by hypoxia were found to be resistant to NK cell-mediated lysis, with granzyme B localising within their autophagosomes (117). When hypoxia-related genes were inhibited, granzyme B activity within target cells was increased (117, 118). However, a later study demonstrated that a major effect of autophagy in cancer cells is to inhibit TNFα and TRAIL-induced apoptosis by reducing FADD-dependent caspase-8 activation (120). Thus, it is possible that autophagy can act in multiple ways to inhibit lymphocyte cytotoxicity.

#### Gasdermins

An emerging aspect of resistance to lymphocyte cytotoxicity is evasion of gasdermin-induced pyroptosis. Both granzyme A and B-mediated cleavage of gasdermin B and E, respectively, have been shown to contribute to tumour control by cytotoxic lymphocytes (48, 54). Furthermore, granzyme A-mediated cleavage of gasdermin B also contributes to defence against bacterial infection by NK cells (55). Several mechanisms have been identified which may cause resistance to these gasdermin-mediated cytotoxic pathways. Firstly, reduced expression of both gasdermin B and E have been identified in many cancers (48, 54). In the case of gasdermin E, reduced expression can occur through epigenetic silencing *via* hypermethylation of the promoter region (121, 122). Low expression of both gasdermin B and E is associated with poor survival in various cancers, including breast cancer, bladder cancer, and melanoma (48, 54). Reducing granzyme-induced pyroptosis through silencing of gasdermin E expression in cancer cells has been shown to contribute significantly to the escape of murine tumours from cytotoxic lymphocytes and accelerate tumour growth (48). Similarly, resistance to gasdermin-mediated pyroptosis can occur through the expression of mutated gasdermin. One study found that 20 out of 22 gasdermin E mutations identified within cancer samples were associated with reduced pyroptosis in response to granzyme B (48).

In the context of bacterial infection, pyroptosis has been shown to be inhibited through degradation of gasdermin B (55). Degradation was mediated by a bacterial ubiquitin ligase IpaH7.8 secreted by the gram-negative bacterium, *Shigella flexneri*. IpaH7.8 was shown to ubiquitinate N-terminal gasdermin B after its cleavage by granzyme A, leading to its degradation. Expression of IpaH7.8 significantly constrained the bactericidal activity of NK cells (55).

In summary, target cells may evade granzyme B-mediated apoptosis through inhibition by serpin B9 or degradation by autophagy. In addition, resistance to gasdermin-mediated pyroptosis is emerging as another mechanism by which cells may evade granzyme-mediated cytotoxicity in the context of malignancy and infection. Importantly, all of these mechanisms can have effects beyond granzymes and can affect other pathways of lymphocyte cytotoxicity as well as cell survival in other contexts. Therefore, targeting these pathways may directly impact cancer and could enhance other modes of treatment, such as immune therapies.

### Inhibiting Death Receptor-Mediated Killing

**Figure 3:**
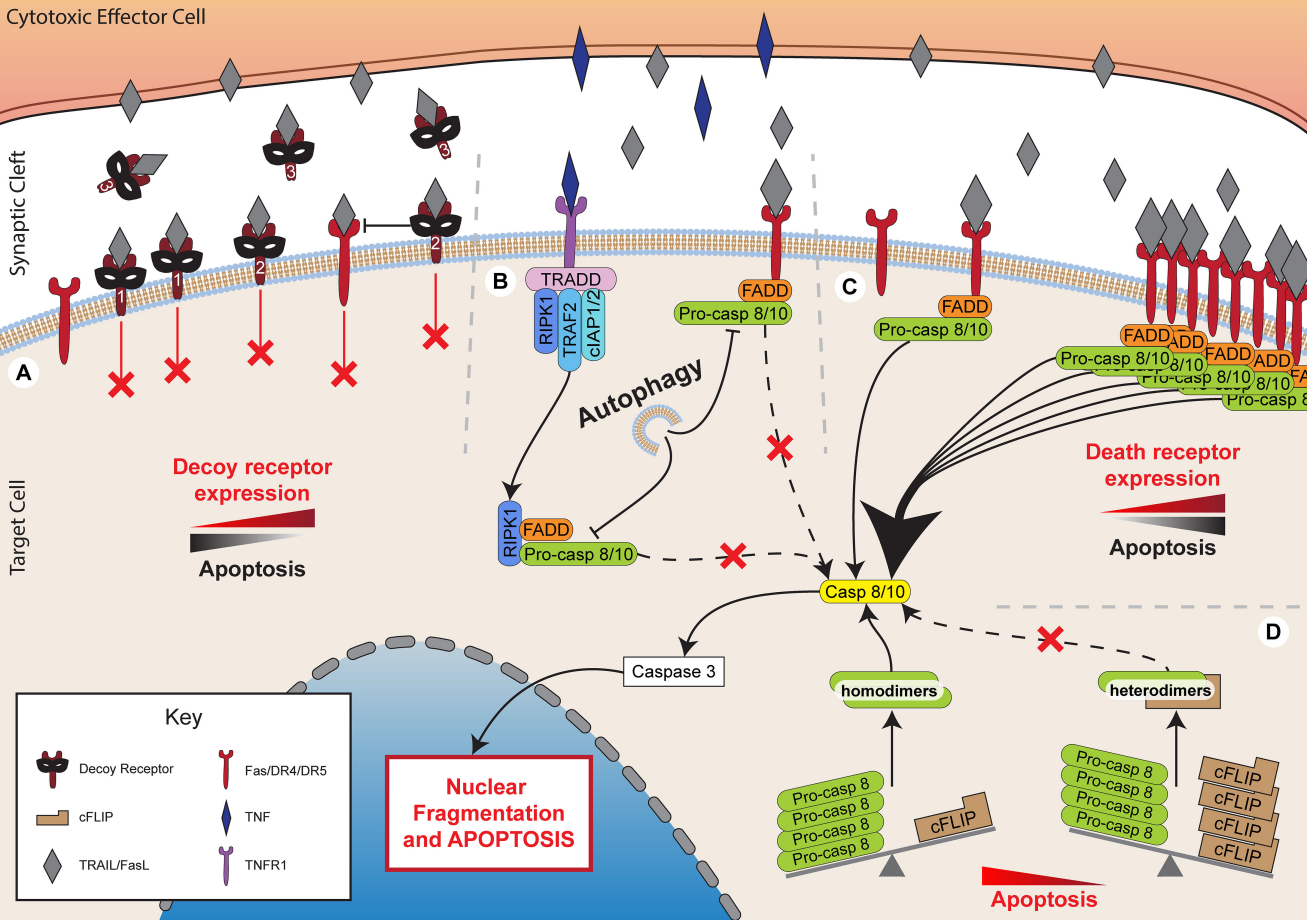
Inhibiting death receptor-mediated killing. Death receptor-mediated killing is a critical method of lymphocyte cytotoxicity. However, target cells have developed multiple mechanisms to inhibit the effectiveness of these processes. **(A)** Expression of decoy receptors, including membrane bound decoy receptors 1 and 2 that lack functional death domains, thereby preventing signalling while sequestering TRAIL. Decoy receptor 2 also inhibits death receptor 5, preventing death receptor 4 recruitment and DISC formation. Decoy receptor 3 is soluble and binds to FasL preventing it acting upon target cell Fas. **(B)** Autophagy inhibits FADD-dependent caspase-8 activation. **(C)** Decreased expression of death receptors, such as Fas, DR4 and DR5 inhibits apoptotic pathways. **(D)** Increased expression of cFLIP sequesters pro-caspase 8 into heterodimers to prevent its cleavage to caspase 8 and the subsequent activation of apoptotic pathways.

Death receptor-mediated cytotoxicity is another critical mechanism by which cytotoxic lymphocytes may eliminate target cells. Several mechanisms have been described by which cells can evade death receptor-mediated cytotoxicity. Signalling can be directly inhibited by the activity of FADD-like IL-1β converting enzyme (FLICE)-inhibitory proteins (FLIPs), expression of decoy receptors, or downregulation of death receptors (**Figure 3**).

#### FLICE Inhibitory Proteins (FLIPs)

One of the best characterised families of death receptor inhibitors are the FLIPs. This family of proteins includes both viral (v-FLIP) and cellular (c-FLIP) proteins, which share high sequence homology (123). Several FLIP splice variants are expressed in humans, but the primary forms include the short variant, c-FLIP_S_, and the long variant, c-FLIP_L_ (123). The long variant contains an additional c-terminal domain that resembles the catalytic domains of caspase 8 and 10 but without functional caspase activity (123, 124).

Both cellular and viral FLIPs inhibit caspase 8 activity by forming heterodimers with pro-caspase 8 (123, 125). This sequesters pro-caspase 8, preventing it from forming the necessary homodimers required for processing into active caspase 8. Inhibition of pro-caspase 8 processing by FLIPs prevents apoptosis induced by TRAIL and FasL, but not by granzyme B (124, 126, 127). Immune cells have been observed to exert a selective pressure on cancer cells during *in vivo* tumorigenesis, allowing cells that highly express FLIP to escape (128). Indeed, high tumour expression of c-FLIP, particularly of the long variant, has been found to correlate with poor prognosis in a range of cancers, including acute myeloid leukaemia, colorectal cancer, and non-small cell lung cancer (129–131).

In addition to cellular FLIPs, viral FLIPs also appear to have a role in the promotion of tumorigenesis in humans. v-FLIPs that are expressed during viral infection act to protect host cells from death receptor-induced apoptosis resulting in immune escape from T cells (132, 133). This viral immune escape mechanism can contribute to the process by which certain viruses are particularly oncogenic. For example, Kaposi’s sarcoma-associated herpesvirus (KSHV)-FLIP is associated with Kaposi’s sarcoma and certain lymphomas (132).

To complicate the picture, although c-FLIP_L_ has primarily been described as an inhibitor of apoptosis, it can also have pro-apoptotic effects. Heterodimers formed of c-FLIP_L_ and pro-caspase 8 have been found to retain their catalytic activity and can process other pro-caspase 8 homodimers (125, 134–137). There is evidence that whether or not c-FLIP_L_ promotes apoptosis is highly dependent on the level of expression of both the FLIP protein and pro-caspase 8 (125, 136, 137). In the presence of very high levels of c-FLIP_L_, and therefore high levels of heterodimers, inhibition of apoptosis occurs since the amount of pro-caspase 8 homodimers that are available to be processed is decreased. Conversely, at lower concentrations, c-FLIP_L_ preferentially acts as a promoter of apoptosis.

Overall, FLIPs may play a significant role in the aetiology of some cancers. As a result, FLIP inhibitors or drugs that reduce FLIP expression are currently under development for the treatment of cancer, but balancing the pro- and anti-apoptotic effects may be challenging (138, 139). Other anti-cancer treatments, such as doxorubicin, synthetic triterpenoids, and peroxisome proliferator-activated receptor-γ (PPARγ) ligands, can also decrease FLIP expression as a side effect, therefore increasing sensitivity to death receptor-mediated cytotoxicity (140–142).

#### Decoy Receptors

The majority of receptors that bind TNF superfamily proteins are capable of transducing signals. However, several receptors have been identified that, despite binding the same ligands, lack cytoplasmic death domains for signalling and cannot recruit critical adaptors, such as FADD (143). These are, in effect, decoy receptors which compete with functional death receptors for ligand binding. Physiologically, decoy receptors have been implicated in modulating inflammatory responses but have also been hijacked as a survival mechanism in cancer (144). One such protein is decoy receptor 3 (DcR3), a soluble receptor which binds FasL and is overexpressed in a large proportion of primary lung, colon, oesophageal, stomach, and rectal tumours (145, 146). Decoy receptors 1 and 2 (DcR1 and DcR2) are membrane-bound receptors which bind TRAIL and also lack a functional death domain (147, 148). In addition to competing with functional TRAIL receptors, DcR2 is also able to interact with TRAIL-receptor variant, DR5, preventing the recruitment of the DR4 variant to the DISC and inhibiting caspase activation (149, 150).

Expression of DcR1 or DcR2 has been found to correlate with tumour progression and poor prognosis in breast cancer, prostate cancer, and leukaemia (151–153). However, it is unclear whether targeting decoy receptors in tumours could have off-target effects, since they can also be expressed in several normal tissues, including the spleen, lung, gastrointestinal tract, endometrium, and activated T cells (145, 154, 155). Furthermore, although there is evidence from over-expression systems that decoy receptors may constrain death receptor-mediated killing, the extent to which they are harnessed by cancer cells to evade lymphocyte cytotoxicity *in vivo* is not known.

#### Death Receptor Expression and Mutation

Death receptor signalling can also be lost in cancer cells through reduced surface expression or through inactivating mutations in death receptors. Reduced expression of the death receptors Fas or DR4/5 is a common feature of cancers (156–158). Loss of these receptors is associated with poor prognosis, particularly upon loss of more than one receptor or when receptor downregulation occurs in tumours with low levels of infiltrating CTL (156, 158). Interestingly, there is a weaker correlation between Fas expression and survival in colorectal tumours with high numbers of infiltrating CTL (158). This may suggest that granule-dependent cytotoxicity rather than death receptor-mediated killing is the predominant pathway of cancer cell elimination when large numbers of CTL are present (158). Loss of death receptor expression can occur through several routes, including promoter methylation (159–161), histone modifications (157, 162), promoter region mutations (163), or reduced trafficking to the cell membrane (164). Notably, oncogenic *Ras* mutations can strongly downregulate Fas expression through the control of several genes associated with the promoter region of *Fas*, as well as through hypermethylation (159, 160). Conversely, death receptors are often up-regulated as a side-effect of cancer treatment, and this may contribute to the overall efficacy of the treatment. For example, receptors for FasL and TRAIL can be significantly up-regulated following radiotherapy and chemotherapy, leading to enhanced killing by cytotoxic lymphocytes (165–171).

A less frequently occurring feature of cancers that may contribute to immune escape is mutations of the death receptors themselves. Mutations affecting function, which generally localise in the cytoplasmic domains, are infrequently observed in cancers, such as gastric cancer, non-small cell lung cancer, metastatic breast cancer, non-Hodgkin’s lymphoma, and head and neck cancer (172–175). Induced expression of these mutated receptors *in vitro* can reduce pro-apoptotic signalling.

Overall, death receptor signalling can be inhibited at multiple stages including reduced expression or mutation of death receptors, competition for ligand binding by decoy receptors, or inhibition of downstream signalling by FLIPs. However, there are several therapeutic strategies that may enhance death receptor signalling including pharmacological inhibition and downregulation of FLIPs or increasing death receptor expression, all of which have the potential to restore the efficacy of immune cytotoxicity.

### Inhibiting TNF-Mediated Cytotoxicity

TNF is known to have both pro-survival and pro-death effects on cancer cells depending on its precise cellular context. Recently, genome-wide CRISPR screens have identified TNF signalling as a major target of resistance to lymphocyte cytotoxicity (120, 176–179). These studies identified several genes encoding proteins related to TNF signalling that either sensitise cells to lymphocyte cytotoxicity – including TNF-R1, caspase 8, TRADD, and RIPK1 – or promote evasion of cytotoxicity – including TRAF2, cIAP1, and FADD-like apoptosis regulator (CFLAR), as well as multiple genes involved in the NF-κB pathway (120, 176–179). In particular, knockout of TRAF2 was shown to redirect TNF signalling from pro-survival signalling, *via* complex I proteins and the NF-κB pathway, to pro-death signalling, *via* complex II proteins (177). Likewise, knockout or pharmacological inhibition of HOIL-1-interacting protein (HOIP), the catalytic subunit of LUBAC involved in ubiquitination, also enhanced sensitivity to TNF by reducing the ubiquitination of pro-survival complex I proteins, which is required for TNF-mediated survival signalling (180, 181). Conversely, antibody blockade of TNF significantly reduced killing by both wild-type and perforin-deficient T cells demonstrating that TNF signalling is a major pathway of lymphocyte cytotoxicity (120, 176).

Resistance to TNF-mediated cell death has also been suggested as one way in which autophagy can induce resistance to lymphocyte cytotoxicity. Knockout of key autophagy-related genes sensitizes cancer cells to TNF-induced death and TNF-mediated T cell cytotoxicity (119, 120). Autophagy can target TNF-induced cell death by modulating FADD/caspase-8 activity (120). Several other studies have also noted the ability of autophagy to interfere with active caspase-8 leading to reduced susceptibility of cells to TRAIL and TNF during hepatic injury or in colon carcinoma (182, 183).

It remains to be seen whether TNF signalling can be harnessed to successfully treat cancer patients in the clinic because its effects are highly context-dependent. This was demonstrated by Young *et al.*, who found that knockout of TNF-R1 could either protect tumours against immune checkpoint blockade or sensitise tumours to it depending on whether autophagy was impaired or intact, respectively (120). TNF has also been shown to have cancer-promoting effects in some cancer models, leading to several clinical trials of TNF antagonists demonstrating efficacy in some patients (184–186). One possible approach to targeting TNF signalling is through the use of SMAC (second mitochondria-derived activator of caspases) mimetics. These are drugs that mimic the activity of SMAC, a protein that is an endogenous inhibitor of cIAP function (187). Through the inhibition of cIAP, SMAC mimetics have been found to sensitise cancer cells to TNF-induced cell death both *in vitro* and *in vivo* (120, 188–190). Alternatively, directly inhibiting certain complex I components, such as HOIP, may redirect signalling towards apoptosis (180). Therefore, there is potential for sensitising cancer cells to TNF-induced cell death, but greater understanding of how its functions vary is needed.

### Inhibiting Apoptotic Pathways

In addition to the inhibitory mechanisms against specific components of lymphocyte cytotoxicity, cancer cells may exhibit more general resistance to apoptosis through alterations in apoptotic pathways. Prevention of apoptosis may occur through either down-regulation of pro-apoptotic mediators, such as caspases or pro-apoptotic Bcl-2 family members, or up-regulation of apoptosis inhibitors, such as Inhibitor of Apoptosis Proteins (IAPs) or anti-apoptotic Bcl-2 family members. These can affect both caspase-dependent and mitochondrial pathways of apoptosis, which are involved in both granule-mediated and death receptor-mediated cell death.

#### Caspase Inhibition – Mutations and Inhibitor of Apoptosis Proteins

Caspases are critical components of both granule-mediated and death receptor-mediated cytotoxicity. Death receptor-induced apoptosis relies on the activation of caspase 8/10 within the DISC to activate the executioner caspases, caspase 3, 6, and 7. Conversely, granzyme B can directly cleave and activate executioner caspases as well as activating them through the mitochondrial pathway of apoptosis. As a result, reducing caspase activity is a common pathway by which cancers avoid apoptosis, with reduced expression or mutations reported for both initiator (caspase 2, 8, and 10) and executioner caspases (caspase 3, 6, and 7) in a range of cancers (191). For example, caspase 8 is commonly mutated, particularly in cancers of neuroendocrine or lymphoid origin (192). Loss of caspase 8 expression contributes to resistance against TRAIL-induced apoptosis (193, 194). In addition to alterations in expression, caspase activity can be modulated by the enhanced expression of IAPs, such as cIAP1, survivin, and X-linked inhibitor of apoptosis protein (XIAP). IAPs can bind directly to caspases preventing their activity and leading to inhibition of granzyme B and death receptor-mediated apoptosis (195–197).

#### Bcl-2 Family

The mitochondrial pathway of apoptosis, characterised by mitochondrial outer membrane permeabilization (MOMP), is a critical pathway by which both granzyme B and death receptors can mediate apoptosis. This pathway is regulated by the Bcl-2 family of proteins, which include pro-apoptotic BH3 proteins (e.g. Bid), pro-apoptotic effector proteins (e.g. Bax and Bak), and anti-apoptotic proteins (e.g. Bcl-2 and Bcl-XL) (40). Disruption of this pathway through either downregulation of pro-apoptotic proteins or upregulation of anti-apoptotic proteins can prevent apoptosis induced by either death receptors or granzymes.

A critical mediator of MOMP is Bid, which can be cleaved by either caspase 8 following death receptor ligation or by granzyme B. Bid is responsible for recruiting additional mediators of MOMP, such as Bax and Bak (40). As a result, loss of Bid expression in cancer cells leads to reduced sensitivity to granzyme B-induced apoptosis (198, 199). Likewise, loss of Bak expression, which is directly involved in permeabilization of the mitochondrial membrane, protects against apoptosis triggered by granzyme B (199). Reduced expression of pro-apoptotic Bcl-2 family members, such as Bid, is observed in various cancers and is associated with poor prognosis in prostate cancer and colon cancer, for example (200, 201).

Alternatively, inhibition of MOMP can occur through overexpression of the anti-apoptotic Bcl-2 family proteins, which inhibit the activity of apoptotic proteins, such as Bax and Bak. Overexpression of anti-apoptotic proteins, such as Bcl-2 or Bcl-XL, reduces apoptosis induced by both granule-mediated cytotoxicity and death receptor-mediated cytotoxicity, whereas pharmacological inhibition of Bcl-2 sensitises cells to cytotoxicity (196, 197, 202–206). Overexpression of Bcl-2 is a common feature of multiple cancers (207).

Overall, cancer cells frequently develop mutations or altered expression of the critical caspases and Bcl-2 family members involved in regulating and mediating apoptosis induced by immune attack. As a result, targeting these pathways, for example by inhibition of Bcl-2, could be a particularly effective way of enhancing the efficacy of immunotherapy in patients (208).

## Conclusion

Effective cytotoxicity by immune cells against cancerous or infected cells is a critical mechanism of controlling these disease states. A multitude of treatments, such as checkpoint inhibitors, have been developed to boost the immune system’s response to these diseased cells. However, these treatments are not always effective, and malignant and infected cells can still exploit mechanisms that enable them to evade the strengthened immune system. Here, we have outlined many ways in which diseased cells can evade the cytotoxic attacks of NK cells and CTLs. Many processes that enhance target cell resistance to cytotoxicity are the same processes that cytotoxic cells themselves use to prevent self-harm by their own deadly cargo. Other pathways that inhibit cytotoxic killing, such as autophagy, pro-survival TNF signalling, and downregulation of apoptotic pathways, also convey other benefits normally, but can be exploited by cancerous cells or infectious agents.

It is likely that many of these mechanisms of resistance have evolved together with the cytotoxic pathways employed by lymphocytes. This may explain why lymphocytes simultaneously utilise several cytotoxic pathways that are often redundant. For example, the broad range of granzyme B targets – including apoptotic caspases, regulators of mitochondrial apoptosis, and gasdermins – reduces the likelihood that a target cell could become resistant to granzyme B-mediated cell death. This was found to be the case in the context of haematological cancer, in which NK cells and CTLs were able to kill cancer cells despite a variety of anti-apoptotic mutations that conferred multi-drug resistance (209, 210). These studies demonstrate that lymphocytes can overcome some resistance mechanisms by inducing death through multiple pathways.

Conceptually, it can be difficult to distinguish processes that have been autonomously selected to aid the survival of diseased cells that also happen to be beneficial in avoiding immune attack, from processes that have been adopted by these cells to specifically resist cell-mediated cytotoxicity. Further research to investigate this could include studying the evolutionary development of cancers, and comparisons with mice deficient in specific compartments of their immune system. Either way, there is great potential here to therapeutically target the processes discussed throughout this review. Targeting the ways in which diseased cells avoid death could be used alone or in combination with other therapies, including immunotherapies. Importantly, a greater understanding of these mechanisms and processes *in vivo* is sorely needed to indicate the most potent interventions.

## Author Contributions

KT wrote the original manuscript. KT and ARA prepared figures. KT, ARA, and DMD edited the manuscript. All authors contributed to the article and approved the submitted version.

## Funding

This work was funded by Cancer Research UK *via* funding to the Cancer Research UK Manchester Institute (to KT; C147/A25254) and a Wellcome Trust Investigator Award (to DMD; 110091/Z/15/Z).

## Conflict of Interest

The authors declare that the research was conducted in the absence of any commercial or financial relationships that could be construed as a potential conflict of interest.

DMD receives research funding from GSK, Continuum Life Sciences and Bristol Myers Squibb, and advises GSK, Mogrify and Bicycle Therapeutics.

## Publisher’s Note

All claims expressed in this article are solely those of the authors and do not necessarily represent those of their affiliated organizations, or those of the publisher, the editors and the reviewers. Any product that may be evaluated in this article, or claim that may be made by its manufacturer, is not guaranteed or endorsed by the publisher.

## References

[1] Prager I Watzl C. Mechanisms of Natural Killer Cell-Mediated Cellular Cytotoxicity. J Leukoc Biol (2019) 105:1319–29. doi: 10.1002/JLB.MR0718-269R 31107565

[2] Topham NJ Hewitt EW. Natural Killer Cell Cytotoxicity: How do They Pull the Trigger? Immunology (2009) 128:7–15. doi: 10.1111/j.1365-2567.2009.03123.x 19689731 PMC2747134

[3] Halle S Halle O Förster R. Mechanisms and Dynamics of T Cell-Mediated Cytotoxicity *In Vivo* . Trends Immunol (2017) 38:432–43. doi: 10.1016/j.it.2017.04.002 28499492

[4] Webster JD Vucic D. The Balance of TNF Mediated Pathways Regulates Inflammatory Cell Death Signaling in Healthy and Diseased Tissues. Front Cell Dev Biol (2020) 8:365. doi: 10.3389/fcell.2020.00365 32671059 PMC7326080

[5] Galluzzi L Vitale I Aaronson SA Abrams JM Adam D Agostinis P. Molecular Mechanisms of Cell Death: Recommendations of the Nomenclature Committee on Cell Death 2018. Cell Death Differ (2018) 25:486–541. doi: 10.1038/s41418-017-0012-4 29362479 PMC5864239

[6] Waldman AD Fritz JM Lenardo MJ. A Guide to Cancer Immunotherapy: From T Cell Basic Science to Clinical Practice. Nat Rev Immunol (2020) 20:651–68. doi: 10.1038/s41577-020-0306-5 PMC7238960 32433532

[7] Chan CJ Smyth MJ Martinet L. Molecular Mechanisms of Natural Killer Cell Activation in Response to Cellular Stress. Cell Death Differ (2014) 21:5–14. doi: 10.1038/cdd.2013.26 23579243 PMC3857624

[8] Davis DM Chiu I Fassett M Cohen GB Mandelboim O Strominger JL. The Human Natural Killer Cell Immune Synapse. Proc Natl Acad Sci (1999) 96:15062–7. doi: 10.1073/pnas.96.26.15062 PMC24773 10611338

[9] Orange JS. Formation and Function of the Lytic NK-Cell Immunological Synapse. Nat Rev Immunol (2008) 8:713–25. doi: 10.1038/nri2381 PMC2772177 19172692

[10] Dustin ML. T-Cell Activation Through Immunological Synapses and Kinapses. Immunol Rev (2008) 221:77–89. doi: 10.1111/j.1600-065X.2008.00589.x 18275476

[11] Stinchcombe JC Bossi G Booth S Griffiths GM. The Immunological Synapse of CTL Contains a Secretory Domain and Membrane Bridges. Immunity (2001) 15:751–61. doi: 10.1016/S1074-7613(01)00234-5 11728337

[12] Monks CRF Freiberg BA Kupfer H Sciaky N Kupfer A. Three-Dimensional Segregation of Supramolecular Activation Clusters in T Cells. Nature (1998) 395:82–6. doi: 10.1038/25764 9738502

[13] Bryceson YT March ME Barber DF Ljunggren H-G Long EO. Cytolytic Granule Polarization and Degranulation Controlled by Different Receptors in Resting NK Cells. J Exp Med (2005) 202:1001–12. doi: 10.1084/jem.20051143 PMC2213171 16203869

[14] Davis DM Dustin ML. What is the Importance of the Immunological Synapse? Trends Immunol (2004) 25:323–7. doi: 10.1016/j.it.2004.03.007 15145322

[15] Kalbasi A Ribas A. Tumour-Intrinsic Resistance to Immune Checkpoint Blockade. Nat Rev Immunol (2020) 20:25–39. doi: 10.1038/s41577-019-0218-4 31570880 PMC8499690

[16] Dhatchinamoorthy K Colbert JD Rock KL. Cancer Immune Evasion Through Loss of MHC Class I Antigen Presentation. Front Immunol (2021) 12:636568. doi: 10.3389/fimmu.2021.636568 33767702 PMC7986854

[17] Spranger S Gajewski TF. Mechanisms of Tumor Cell–Intrinsic Immune Evasion. Annu Rev Cancer Biol (2018) 2:213–28. doi: 10.1146/annurev-cancerbio-030617-050606

[18] O’Donnell JS Teng MWL Smyth MJ. Cancer Immunoediting and Resistance to T Cell-Based Immunotherapy. Nat Rev Clin Oncol (2019) 16:151–67. doi: 10.1038/s41571-018-0142-8 30523282

[19] Ribas A Wolchok JD. Cancer Immunotherapy Using Checkpoint Blockade. Science (2018) 359:1350–5. doi: 10.1126/science.aar4060 PMC7391259 29567705

[20] Hanahan D Weinberg RA. Hallmarks of Cancer: The Next Generation. Cell (2011) 144:646–74. doi: 10.1016/j.cell.2011.02.013 21376230

[21] Dustin ML. The Immunological Synapse. Cancer Immunol Res (2014) 2:1023–33. doi: 10.1158/2326-6066.CIR-14-0161 PMC4692051 25367977

[22] Mace EM Dongre P Hsu H-T Sinha P James AM Mann SS. Cell Biological Steps and Checkpoints in Accessing NK Cell Cytotoxicity. Immunol Cell Biol (2014) 92:245–55. doi: 10.1038/icb.2013.96 PMC3960583 24445602

[23] Lagrue K Carisey A Oszmiana A Kennedy PR Williamson DJ Cartwright A. The Central Role of the Cytoskeleton in Mechanisms and Functions of the NK Cell Immune Synapse. Immunol Rev (2013) 256:203–21. doi: 10.1111/imr.12107 24117823

[24] Osińska I Popko K Demkow U. Perforin: An Important Player in Immune Response. Cent J Immunol (2014) 39:109–15. doi: 10.5114/ceji.2014.42135 PMC4439970 26155110

[25] Chowdhury D Lieberman J. Death by a Thousand Cuts: Granzyme Pathways of Programmed Cell Death. Annu Rev Immunol (2008) 26:389–420. doi: 10.1146/annurev.immunol.26.021607.090404 18304003 PMC2790083

[26] Voskoboinik I Whisstock JC Trapani JA. Perforin and Granzymes: Function, Dysfunction and Human Pathology. Nat Rev Immunol (2015) 15:388–400. doi: 10.1038/nri3839 25998963

[27] Backes CS Friedmann KS Mang S Knörck A Hoth M Kummerow C. Natural Killer Cells Induce Distinct Modes of Cancer Cell Death: Discrimination, Quantification, and Modulation of Apoptosis, Necrosis, and Mixed Forms. J Biol Chem (2018) 293:16348–63. http://www.jbc.org/content/293/42/16348.abstract. doi: 10.1074/jbc.RA118.004549 PMC6200954 30190323

[28] Masson D Tschopp J. Isolation of a Lytic, Pore-Forming Protein (Perforin) From Cytolytic T-Lymphocytes. J Biol Chem (1985) 260:9069–72. doi: 10.1016/S0021-9258(17)39328-6 3874868

[29] Law RHP Lukoyanova N Voskoboinik I Caradoc-Davies TT Baran K Dunstone MA. The Structural Basis for Membrane Binding and Pore Formation by Lymphocyte Perforin. Nature (2010) 468:447. doi: 10.1038/nature09518 21037563

[30] Lopez JA Susanto O Jenkins MR Lukoyanova N Sutton VR Law RHP. Perforin Forms Transient Pores on the Target Cell Plasma Membrane to Facilitate Rapid Access of Granzymes During Killer Cell Attack. Blood (2013) 121:2659–68. doi: 10.1182/blood-2012-07-446146 23377437

[31] Thiery J Keefe D Boulant S Boucrot E Walch M Martinvalet D. Perforin Pores in the Endosomal Membrane Trigger the Release of Endocytosed Granzyme B Into the Cytosol of Target Cells. Nat Immunol (2011) 12:770. doi: 10.1038/ni.2050 21685908 PMC3140544

[32] Keefe D Shi L Feske S Massol R Navarro F Kirchhausen T. Perforin Triggers a Plasma Membrane-Repair Response That Facilitates CTL Induction of Apoptosis. Immunity (2005) 23:249–62. doi: 10.1016/j.immuni.2005.08.001 16169498

[33] Thiery J Keefe D Saffarian S Martinvalet D Walch M Boucrot E. Perforin Activates Clathrin- and Dynamin-Dependent Endocytosis, Which is Required for Plasma Membrane Repair and Delivery of Granzyme B for Granzyme-Mediated Apoptosis. Blood (2010) 115:1582–93. doi: 10.1182/blood-2009-10-246116 PMC2830763 20038786

[34] Ambrose AR Hazime KS Worboys JD Niembro-Vivanco O Davis DM. Synaptic Secretion From Human Natural Killer Cells is Diverse and Includes Supramolecular Attack Particles. Proc Natl Acad Sci (2020) 117:23717–20. doi: 10.1073/pnas.2010274117 PMC7519227 32900953

[35] Bálint Š Müller S Fischer R Kessler BM Harkiolaki M Valitutti S. Supramolecular Attack Particles are Autonomous Killing Entities Released From Cytotoxic T Cells. Science (80-) (2020) 368:897–901. doi: 10.1126/science.aay9207 PMC7116847 32381591

[36] Elmore S. Apoptosis: A Review of Programmed Cell Death. Toxicol Pathol (2007) 35:495–516. doi: 10.1080/01926230701320337 17562483 PMC2117903

[37] Sutton VR Davis JE Cancilla M Johnstone RW Ruefli AA Sedelies K. Initiation of Apoptosis by Granzyme B Requires Direct Cleavage of Bid, But Not Direct Granzyme B–Mediated Caspase Activation. J Exp Med (2000) 192:1403–14. doi: 10.1084/jem.192.10.1403 PMC2193191 11085743

[38] Catalán E Jaime-Sánchez P Aguiló N Simon MM Froelich CJ Pardo J. Mouse Cytotoxic T Cell-Derived Granzyme B Activates the Mitochondrial Cell Death Pathway in a Bim-Dependent Fashion *. J Biol Chem (2015) 290:6868–77. doi: 10.1074/jbc.M114.631564 PMC4358112 25605735

[39] Pardo J Wallich R Martin P Urban C Rongvaux A Flavell RA. Granzyme B-Induced Cell Death Exerted by Ex Vivo CTL: Discriminating Requirements for Cell Death and Some of its Signs. Cell Death Differ (2008) 15:567–79. doi: 10.1038/sj.cdd.4402289 18064039

[40] Kalkavan H Green DR. MOMP. Cell Suicide as a BCL-2 Family Business. Cell Death Differ (2018) 25:46–55. doi: 10.1038/cdd.2017.179 29053143 PMC5729535

[41] Han J Goldstein LA Gastman BR Froelich CJ Yin X-M Rabinowich H. Degradation of Mcl-1 by Granzyme B: IMPLICATIONS FOR Bim-MEDIATED MITOCHONDRIAL APOPTOTIC EVENTS *. J Biol Chem (2004) 279:22020–9. doi: 10.1074/jbc.M313234200 15014070

[42] Thomas DA Scorrano L Putcha GV Korsmeyer SJ Ley TJ. Granzyme B can Cause Mitochondrial Depolarization and Cell Death in the Absence of BID, BAX, and BAK. Proc Natl Acad Sci (2001) 98:14985. doi: 10.1073/pnas.261581498 11752447 PMC64970

[43] Goping IS Sawchuk T Rieger A Shostak I Bleackley RC. Cytotoxic T Lymphocytes Overcome Bcl-2 Inhibition: Target Cells Contribute to Their Own Demise. Blood (2008) 111:2142–51. doi: 10.1182/blood-2007-08-105221 18096765

[44] Martinvalet D. Mitochondrial Entry of Cytotoxic Proteases: A New Insight Into the Granzyme B Cell Death Pathway. Oxid Med Cell Longev (2019) 2019:9165214. doi: 10.1155/2019/9165214 31249651 PMC6556269

[45] Thomas DA Du C Xu M Wang X Ley TJ. DFF45/ICAD Can Be Directly Processed by Granzyme B During the Induction of Apoptosis. Immunity (2000) 12:621–32. doi: 10.1016/S1074-7613(00)80213-7 10894162

[46] Adrain C Duriez PJ Brumatti G Delivani P Martin SJ. The Cytotoxic Lymphocyte Protease, Granzyme B, Targets the Cytoskeleton and Perturbs Microtubule Polymerization Dynamics *. J Biol Chem (2006) 281:8118–25. doi: 10.1074/jbc.M509361200 16415351

[47] Goping IS Sawchuk T Underhill DA Bleackley RC. Identification of α-Tubulin as a Granzyme B Substrate During CTL-Mediated Apoptosis. J Cell Sci (2006) 119:858–65. doi: 10.1242/jcs.02791 16495481

[48] Zhang Z Zhang Y Xia S Kong Q Li S Liu X. Gasdermin E Suppresses Tumour Growth by Activating Anti-Tumour Immunity. Nature (2020) 579:415–20. doi: 10.1038/s41586-020-2071-9 PMC7123794 32188940

[49] Yuying L Yiliang F Xinfeng C Zhenfeng W Xiaoyu L Tianzhen Z. Gasdermin E–mediated Target Cell Pyroptosis by CAR T Cells Triggers Cytokine Release Syndrome. Sci Immunol (2020) 5:eaax7969. doi: 10.1126/sciimmunol.aax7969 31953257

[50] Liu X Xia S Zhang Z Wu H Lieberman J. Channelling Inflammation: Gasdermins in Physiology and Disease. Nat Rev Drug Discovery (2021) 20:384–405. doi: 10.1038/s41573-021-00154-z 33692549 PMC7944254

[51] Mahrus S Craik CS. Selective Chemical Functional Probes of Granzymes A and B Reveal Granzyme B Is a Major Effector of Natural Killer Cell-Mediated Lysis of Target Cells. Chem Biol (2005) 12:567–77. doi: 10.1016/j.chembiol.2005.03.006 15911377

[52] Beresford PJ Xia Z Greenberg AH Lieberman J. Granzyme A Loading Induces Rapid Cytolysis and a Novel Form of DNA Damage Independently of Caspase Activation. Immunity (1999) 10:585–95. doi: 10.1016/S1074-7613(00)80058-8 10367904

[53] Lieberman J. Granzyme A Activates Another Way to Die. Immunol Rev (2010) 235:93–104. doi: 10.1111/j.0105-2896.2010.00902.x 20536557 PMC2905780

[54] Zhou Z He H Wang K Shi X Wang Y Su Y. Granzyme A From Cytotoxic Lymphocytes Cleaves GSDMB to Trigger Pyroptosis in Target Cells. Science (80-) (2020) 388:eaaz7548. doi: 10.1126/science.aaz7548 32299851

[55] Hansen JM de Jong MF Wu Q Zhang L-S Heisler DB Alto LT. Pathogenic Ubiquitination of GSDMB Inhibits NK Cell Bactericidal Functions. Cell (2021) 184:3178–3191.e18. doi: 10.1016/j.cell.2021.04.036 34022140 PMC8221529

[56] Wilson NS Dixit V Ashkenazi A. Death Receptor Signal Transducers: Nodes of Coordination in Immune Signaling Networks. Nat Immunol (2009) 10:348–55. doi: 10.1038/ni.1714 19295631

[57] Bodmer J-L Schneider P Tschopp J. The Molecular Architecture of the TNF Superfamily. Trends Biochem Sci (2002) 27:19–26. doi: 10.1016/S0968-0004(01)01995-8 11796220

[58] Micheau O Tschopp J. Induction of TNF Receptor I-Mediated Apoptosis *via* Two Sequential Signaling Complexes. Cell (2003) 114:181–90. doi: 10.1016/S0092-8674(03)00521-X 12887920

[59] Wang L Du F Wang X. TNF-α Induces Two Distinct Caspase-8 Activation Pathways. Cell (2008) 133:693–703. doi: 10.1016/j.cell.2008.03.036 18485876

[60] Bertrand MJM Milutinovic S Dickson KM Ho WC Boudreault A Durkin J. Ciap1 and Ciap2 Facilitate Cancer Cell Survival by Functioning as E3 Ligases That Promote RIP1 Ubiquitination. Mol Cell (2008) 30:689–700. doi: 10.1016/j.molcel.2008.05.014 18570872

[61] Weigelin B den Boer AT Wagena E Broen K Dolstra H de Boer RJ. Cytotoxic T Cells are Able to Efficiently Eliminate Cancer Cells by Additive Cytotoxicity. Nat Commun (2021) 12:5217. doi: 10.1038/s41467-021-25282-3 34471116 PMC8410835

[62] Khazen R Cazaux M Lemaître F Corre B Garcia Z Bousso P. Functional Heterogeneity of Cytotoxic T Cells and Tumor Resistance to Cytotoxic Hits Limit Anti-Tumor Activity *In Vivo* . EMBO J (2021) 40:e106658. doi: 10.15252/embj.2020106658 33855732 PMC8167356

[63] Liu CC Jiang S Persechini PM Zychlinsky A Kaufmann Y Young JD. Resistance of Cytolytic Lymphocytes to Perforin-Mediated Killing. Induction of Resistance Correlates With Increase in Cytotoxicity. J Exp Med (1989) 169:2211–25. doi: 10.1084/jem.169.6.2211 PMC2189341 2786549

[64] Shinkai Y Takio K Okumura K. Homology of Perforin to the Ninth Component of Complement (C9). Nature (1988) 334:525–7. doi: 10.1038/334525a0 3261391

[65] Jiang SB Ojcius DM Persechini PM Young JD. Resistance of Cytolytic Lymphocytes to Perforin-Mediated Killing. Inhibition of Perforin Binding Activity by Surface Membrane Proteins. J Immunol (1990) 144:998–1003. 2104915

[66] Lehmann C Zeis M Schmitz N Uharek L. Impaired Binding of Perforin on the Surface of Tumor Cells is a Cause of Target Cell Resistance Against Cytotoxic Effector Cells(2000). 10887123

[67] Otten HG van Ginkel WGJ Hagenbeek A Petersen EJ. Prevalence and Clinical Significance of Resistance to Perforin- and FAS-Mediated Cell Death in Leukemia. Leukemia (2004) 18:1401–5. doi: 10.1038/sj.leu.2403414 15215873

[68] Tuomela K Mukherjee D Ambrose AR Harikrishnan A Mole H Hurlstone A. Radiotherapy Transiently Reduces the Sensitivity of Cancer Cells to Lymphocyte Cytotoxicity. Proc Natl Acad Sci (2022) 119:e2111900119. doi: 10.1073/pnas.2111900119 35042775 PMC8785960

[69] Owen DM Rentero C Magenau A Abu-Siniyeh A Gaus K. Quantitative Imaging of Membrane Lipid Order in Cells and Organisms. Nat Protoc (2012) 7:24–35. doi: 10.1038/nprot.2011.419 22157973

[70] Simons K Vaz WLC. Model Systems, Lipid Rafts, and Cell Membranes. Annu Rev Biophys Biomol Struct (2004) 33:269–95. doi: 10.1146/annurev.biophys.32.110601.141803 15139814

[71] Blumenthal R Millard PJ Henkart MP Reynolds CW Henkart PA. Liposomes as Targets for Granule Cytolysin From Cytotoxic Large Granular Lymphocyte Tumors. Proc Natl Acad Sci USA (1984) 81:5551–5. doi: 10.1073/pnas.81.17.5551 PMC391744 6591203

[72] Antia R Schlegel RA Williamson P. Binding of Perforin to Membranes is Sensitive to Lipid Spacing and Not Headgroup. Immunol Lett (1992) 32:153–7. doi: 10.1016/0165-2478(92)90108-Z 1612639

[73] Rudd-Schmidt JA Hodel AW Noori T Lopez JA Cho H-J Verschoor S. Lipid Order and Charge Protect Killer T Cells From Accidental Death. Nat Commun (2019) 10:5396. doi: 10.1038/s41467-019-13385-x 31776337 PMC6881447

[74] Hodel AW Rudd-Schmidt J Trapani JA Voskoboinik I Hoogenboom B. Lipid Specificity of the Immune Effector Perforin. Faraday Discuss (2020) 232:236–55. doi: 10.1039/D0FD00043D PMC8704153 34545865

[75] Li Y Orange JS. Degranulation Enhances Presynaptic Membrane Packing, Which Protects NK Cells From Perforin-Mediated Autolysis. PLoS Biol (2021) 19:e3001328. doi: 10.1371/journal.pbio.3001328 34343168 PMC8330931

[76] Gaus K Chklovskaia E Fazekas de St. Groth B Jessup W Harder T. Condensation of the Plasma Membrane at the Site of T Lymphocyte Activation. J Cell Biol (2005) 171:121–31. doi: 10.1083/jcb.200505047 PMC2171224 16203859

[77] Janes PW Ley SC Magee AI. Aggregation of Lipid Rafts Accompanies Signaling *via* the T Cell Antigen Receptor. J Cell Biol (1999) 147:447–61. doi: 10.1083/jcb.147.2.447 PMC2174214 10525547

[78] Baritaki S Apostolakis S Kanellou P Dimanche-Boitrel M Spandidos DA. Bonavida BBT-A in CR. Reversal of Tumor Resistance to Apoptotic Stimuli by Alteration of Membrane Fluidity: Therapeutic Implications. Adv Cancer Res (2007) 98:149–90. doi: 10.1016/S0065-230X(06)98005-1 17433910

[79] Zalba S ten Hagen TLM. Cell Membrane Modulation as Adjuvant in Cancer Therapy. Cancer Treat Rev (2017) 52:48–57. doi: 10.1016/j.ctrv.2016.10.008 27889637 PMC5195909

[80] Kay JG Fairn GD. Distribution, Dynamics and Functional Roles of Phosphatidylserine Within the Cell. Cell Commun Signal (2019) 17:126. doi: 10.1186/s12964-019-0438-z 31615534 PMC6792266

[81] Shin H-W Takatsu H. Phosphatidylserine Exposure in Living Cells. Crit Rev Biochem Mol Biol (2020) 55:166–78. doi: 10.1080/10409238.2020.1758624 32408772

[82] Fischer K Voelkl S Berger J Andreesen R Pomorski T Mackensen A. Antigen Recognition Induces Phosphatidylserine Exposure on the Cell Surface of Human CD8+ T Cells. Blood (2006) 108:4094–101. doi: 10.1182/blood-2006-03-011742 16912227

[83] Riedl S Rinner B Asslaber M Schaider H Walzer S Novak A. In Search of a Novel Target — Phosphatidylserine Exposed by Non-Apoptotic Tumor Cells and Metastases of Malignancies With Poor Treatment Efficacy. Biochim Biophys Acta - Biomembr (2011) 1808:2638–45. doi: 10.1016/j.bbamem.2011.07.026 PMC3175029 21810406

[84] Hernández-Castañeda MA Lavergne M Casanova P Nydegger B Merten C Subramanian BY. A Profound Membrane Reorganization Defines Susceptibility of Plasmodium Falciparum Infected Red Blood Cells to Lysis by Granulysin and Perforin. Front Immunol (2021) 12:643746. doi: 10.3389/fimmu.2021.643746 34093532 PMC8170093

[85] Suresh S. Biomechanics and Biophysics of Cancer Cells. Acta Mater (2007) 55:3989–4014. doi: 10.1016/j.actamat.2007.04.022 PMC2917191 17540628

[86] Liu Y Zhang T Zhang H Li J Zhou N Fiskesund R. Cell Softness Prevents Cytolytic T-Cell Killing of Tumor-Repopulating Cells. Cancer Res (2021) 81:476–88. doi: 10.1158/0008-5472.CAN-20-2569 33168645

[87] Basu R Whitlock BM Husson J Le Floc’h A Jin W Oyler-Yaniv A. Cytotoxic T Cells Use Mechanical Force to Potentiate Target Cell Killing. Cell (2016) 165:100–10. doi: 10.1016/j.cell.2016.01.021 PMC4808403 26924577

[88] Lee M-T Chen F-Y Huang HW. Energetics of Pore Formation Induced by Membrane Active Peptides. Biochemistry (2004) 43:3590–9. doi: 10.1021/bi036153r 15035629

[89] Tamzalit F Wang MS Jin W Tello-Lafoz M Boyko V Heddleston JM. Interfacial Actin Protrusions Mechanically Enhance Killing by Cytotoxic T Cells. Sci Immunol (2019) 4:eaav5445. doi: 10.1126/sciimmunol.aav5445 30902904 PMC6746172

[90] Friedman D Simmonds P Hale A Bere L Hodson NW White MRH. Natural Killer Cell Immune Synapse Formation and Cytotoxicity are Controlled by Tension of the Target Interface. J Cell Sci (2021) 134:jcs258570. doi: 10.1242/jcs.258570 33712452 PMC8077183

[91] Tello-Lafoz M Srpan K Sanchez EE Hu J Remsik J Romin Y. Cytotoxic Lymphocytes Target Characteristic Biophysical Vulnerabilities in Cancer. Immunity (2021) 54:1037–54.e7. doi: 10.1016/j.immuni.2021.02.020 33756102 PMC8119359

[92] Perišić Nanut M Sabotič J Jewett A Kos J. Cysteine Cathepsins as Regulators of the Cytotoxicity of NK and T Cells. Front Immunol (2014) 5:616. doi: 10.3389/fimmu.2014.00616 25520721 PMC4251435

[93] Balaji KN Schaschke N Machleidt W Catalfamo M Henkart PA. Surface Cathepsin B Protects Cytotoxic Lymphocytes From Self-Destruction After Degranulation. J Exp Med (2002) 196:493–503. doi: 10.1084/jem.20011836 12186841 PMC2196055

[94] Baran K Ciccone A Peters C Yagita H Bird PI Villadangos JA. Cytotoxic T Lymphocytes From Cathepsin B-Deficient Mice Survive Normally *In Vitro* and *In Vivo* After Encountering and Killing Target Cells. J Biol Chem (2006) 281:30485–91. doi: 10.1074/jbc.M602007200 16914553

[95] Khazen R Müller S Gaudenzio N Espinosa E Puissegur M-P Valitutti S. Melanoma Cell Lysosome Secretory Burst Neutralizes the CTL-Mediated Cytotoxicity at the Lytic Synapse. Nat Commun (2016) 7:10823. doi: 10.1038/ncomms10823 26940455 PMC4785227

[96] Ruan H Hao S Young P Zhang H. Targeting Cathepsin B for Cancer Therapies. Horizons Cancer Res (2015) 56:23–40. PMC4662557 26623174

[97] Al Absi A Wurzer H Guerin C Hoffmann C Moreau F Mao X. Actin Cytoskeleton Remodeling Drives Breast Cancer Cell Escape From Natural Killer–Mediated Cytotoxicity. Cancer Res (2018) 78:5631–43. doi: 10.1158/0008-5472.CAN-18-0441 30104240

[98] Wurzer H Filali L Hoffmann C Krecke M Biolato AM Mastio J. Intrinsic Resistance of Chronic Lymphocytic Leukemia Cells to NK Cell-Mediated Lysis Can Be Overcome In Vitro by Pharmacological Inhibition of Cdc42-Induced Actin Cytoskeleton Remodeling. Front Immunol (2021) 12:619069. doi: 10.3389/fimmu.2021.619069 34108958 PMC8181408

[99] Sun J Bird CH Sutton V McDonald L Coughlin PB De Jong TA. A Cytosolic Granzyme B Inhibitor Related to the Viral Apoptotic Regulator Cytokine Response Modifier A Is Present in Cytotoxic Lymphocytes. J Biol Chem (1996) 271:27802–9. doi: 10.1074/jbc.271.44.27802 8910377

[100] Bird CH Sutton VR Sun J Hirst CE Novak A Kumar S. Selective Regulation of Apoptosis: The Cytotoxic Lymphocyte Serpin Proteinase Inhibitor 9 Protects Against Granzyme B-Mediated Apoptosis Without Perturbing the Fas Cell Death Pathway. Mol Cell Biol (1998) 18:6387–98. doi: 10.1128/MCB.18.11.6387 PMC109224 9774654

[101] Sanrattana W Maas C de Maat S. SERPINs—From Trap to Treatment. Front Med (2019) 6:25. doi: 10.3389/fmed.2019.00025 PMC6379291 30809526

[102] Bladergroen BA Meijer CJLM ten Berge RL Hack CE Muris JJF Dukers DF. Expression of the Granzyme B Inhibitor, Protease Inhibitor 9, by Tumor Cells in Patients With Non-Hodgkin and Hodgkin Lymphoma: A Novel Protective Mechanism for Tumor Cells to Circumvent the Immune System? Blood (2002) 99:232–7. doi: 10.1182/BLOOD.V99.1.232 11756176

[103] van Houdt IS Oudejans JJ van den Eertwegh AJM Baars A Vos W Bladergroen BA. Expression of the Apoptosis Inhibitor Protease Inhibitor 9 Predicts Clinical Outcome in Vaccinated Patients With Stage III and IV Melanoma. Clin Cancer Res (2005) 11:6400–7. doi: 10.1158/1078-0432.CCR-05-0306 16144945

[104] Medema JP de Jong J Peltenburg LT Verdegaal EM Gorter A Bres SA. Blockade of the Granzyme B/perforin Pathway Through Overexpression of the Serine Protease Inhibitor PI-9/SPI-6 Constitutes a Mechanism for Immune Escape by Tumors. Proc Natl Acad Sci U S A (2001) 98:11515–20. doi: 10.1073/pnas.201398198 PMC58761 11562487

[105] ten Berge RL Meijer CJLM Dukers DF Kummer JA Bladergroen BA Vos W. Expression Levels of Apoptosis-Related Proteins Predict Clinical Outcome in Anaplastic Large Cell Lymphoma. Blood (2002) 99:4540–6. doi: 10.1182/BLOOD.V99.12.4540 12036886

[106] Godal R Keilholz U Uharek L Letsch A Asemissen AM Busse A. Lymphomas are Sensitive to Perforin-Dependent Cytotoxic Pathways Despite Expression of PI-9 and Overexpression of Bcl-2. Blood (2006) 107:3205–11. doi: 10.1182/blood-2005-07-2880 16373664

[107] Jiang L Wang Y-J Zhao J Uehara M Hou Q Kasinath V. Direct Tumor Killing and Immunotherapy Through Anti-SerpinB9 Therapy. Cell (2020) 183:1219–1233.e18. doi: 10.1016/j.cell.2020.10.045 33242418 PMC7927154

[108] Cunningham TD Jiang X Shapiro DJ. Expression of High Levels of Human Proteinase Inhibitor 9 Blocks Both Perforin/Granzyme and Fas/Fas Ligand-Mediated Cytotoxicity. Cell Immunol (2007) 245:32–41. doi: 10.1016/j.cellimm.2007.03.004 17490628 PMC3655900

[109] Ray M Hostetter DR Loeb CRK Simko J Craik CS. Inhibition of Granzyme B by PI-9 Protects Prostate Cancer Cells From Apoptosis. Prostate (2012) 72:846–55. doi: 10.1002/pros.21486 PMC3401211 21919028

[110] Liesche C Sauer P Prager I Urlaub D Claus M Eils R. Single-Fluorescent Protein Reporters Allow Parallel Quantification of Natural Killer Cell-Mediated Granzyme and Caspase Activities in Single Target Cells. Front Immunol (2018) 9:1840. doi: 10.3389/fimmu.2018.01840 30135688 PMC6092488

[111] Jiang P Gu S Pan D Fu J Sahu A Hu X. Signatures of T Cell Dysfunction and Exclusion Predict Cancer Immunotherapy Response. Nat Med (2018) 24:1550–8. doi: 10.1038/s41591-018-0136-1 PMC6487502 30127393

[112] Chen J Cao Y Markelc B Kaeppler J Vermeer JA Muschel RJ. Type I IFN Protects Cancer Cells From CD8+ T Cell-Mediated Cytotoxicity After Radiation. J Clin Invest (2019) 129:4224–38. doi: 10.1172/JCI127458 PMC6763250 31483286

[113] Choi PJ Mitchison TJ. Quantitative Analysis of Resistance to Natural Killer Attacks Reveals Stepwise Killing Kinetics. Integr Biol (2014) 6:1153–61. doi: 10.1039/c4ib00096j 25228316

[114] Kummer JA Micheau O Schneider P Bovenschen N Broekhuizen R Quadir R. Ectopic Expression of the Serine Protease Inhibitor PI9 Modulates Death Receptor-Mediated Apoptosis. Cell Death Differ (2007) 14:1486–96. doi: 10.1038/sj.cdd.4402152 17479112

[115] Yun CW Lee SH. The Roles of Autophagy in Cancer. Int J Mol Sci (2018) 19:3466. doi: 10.3390/ijms19113466 PMC6274804 30400561

[116] Noman MZ Janji B Kaminska B Van Moer K Pierson S Przanowski P. Blocking Hypoxia-Induced Autophagy in Tumors Restores Cytotoxic T-Cell Activity and Promotes Regression. Cancer Res (2011) 71:5976–86. doi: 10.1158/0008-5472.CAN-11-1094 21810913

[117] Baginska J Viry E Berchem G Poli A Noman MZ van Moer K. Granzyme B Degradation by Autophagy Decreases Tumor Cell Susceptibility to Natural Killer-Mediated Lysis Under Hypoxia. Proc Natl Acad Sci U S A (2013) 110:17450–5. doi: 10.1073/pnas.1304790110 PMC3808626 24101526

[118] Messai Y Noman MZ Hasmim M Janji B Tittarelli A Boutet M. ITPR1 Protects Renal Cancer Cells Against Natural Killer Cells by Inducing Autophagy. Cancer Res (2014) 74:6820–32. doi: 10.1158/0008-5472.CAN-14-0303 25297632

[119] Lawson KA Sousa CM Zhang X Kim E Akthar R Caumanns JJ. Functional Genomic Landscape of Cancer-Intrinsic Evasion of Killing by T Cells. Nature (2020) 586:120–6. doi: 10.1038/s41586-020-2746-2 PMC9014559 32968282

[120] Young TM Reyes C Pasnikowski E Castanaro C Wong C Decker CE. Autophagy Protects Tumors From T Cell-Mediated Cytotoxicity *via* Inhibition of Tnfα-Induced Apoptosis. Sci Immunol (2020) 5:eabb9561. doi: 10.1126/sciimmunol.abb9561 33443027

[121] Kim MS Chang X Yamashita K Nagpal JK Baek JH Wu G. Aberrant Promoter Methylation and Tumor Suppressive Activity of the DFNA5 Gene in Colorectal Carcinoma. Oncogene (2008) 27:3624–34. doi: 10.1038/sj.onc.1211021 18223688

[122] Akino K Toyota M Suzuki H Imai T Maruyama R Kusano M. Identification of DFNA5 as a Target of Epigenetic Inactivation in Gastric Cancer. Cancer Sci (2007) 98:88–95. doi: 10.1111/j.1349-7006.2006.00351.x 17083569 PMC11158324

[123] Humphreys L Espona-Fiedler M Longley DB. FLIP as a Therapeutic Target in Cancer. FEBS J (2018) 285:4104–23. doi: 10.1111/febs.14523 29806737

[124] Hu S Vincenz C Ni J Gentz R Dixit VM. I-FLICE. A Novel Inhibitor of Tumor Necrosis Factor Receptor-1- and CD-95-Induced Apoptosis. J Biol Chem (1997) 272:17255–7. doi: 10.1074/jbc.272.28.17255 9211860

[125] Hughes MA Powley IR Jukes-Jones R Horn S Feoktistova M Fairall L. Co-Operative and Hierarchical Binding of C-FLIP and Caspase-8: A Unified Model Defines How C-FLIP Isoforms Differentially Control Cell Fate. Mol Cell (2016) 61:834–49. doi: 10.1016/j.molcel.2016.02.023 PMC4819448 26990987

[126] Kataoka T Schröter M Hahne M Schneider P Irmler M Thome M. FLIP Prevents Apoptosis Induced by Death Receptors But Not by Perforin/Granzyme B, Chemotherapeutic Drugs, and Gamma Irradiation. J Immunol (1998) 161:3936–42. 9780161

[127] Irmler M Thome M Hahne M Schneider P Hofmann K Steiner V. Inhibition of Death Receptor Signals by Cellular FLIP. Nature (1997) 388:190–5. doi: 10.1038/40657 9217161

[128] Medema JP de Jong J van Hall T Melief CJ Offringa R. Immune Escape of Tumors *In Vivo* by Expression of Cellular FLICE-Inhibitory Protein. J Exp Med (1999) 190:1033–8. doi: 10.1084/jem.190.7.1033 PMC2195655 10510093

[129] Riley JS Hutchinson R McArt DG Crawford N Holohan C Paul I. Prognostic and Therapeutic Relevance of FLIP and Procaspase-8 Overexpression in Non-Small Cell Lung Cancer. Cell Death Dis (2013) 4:e951–1. doi: 10.1038/cddis.2013.481 PMC3877552 24309938

[130] Ullenhag GJ Mukherjee A Watson NFS Al-Attar AH Scholefield JH Durrant LG. Overexpression of FLIP_L_ Is an Independent Marker of Poor Prognosis in Colorectal Cancer Patients. Clin Cancer Res (2007) 13:5070. doi: 10.1158/1078-0432.CCR-06-2547 17785559

[131] McLornan D Hay J McLaughlin K Holohan C Burnett AK Hills RK. Prognostic and Therapeutic Relevance of C-FLIP in Acute Myeloid Leukaemia. Br J Haematol (2013) 160:188–98. doi: 10.1111/bjh.12108 23167276

[132] Djerbi M Screpanti V Catrina AI Bogen B Biberfeld P Grandien A. The Inhibitor of Death Receptor Signaling, FLICE-Inhibitory Protein Defines a New Class of Tumor Progression Factors. J Exp Med (1999) 190:1025–32. doi: 10.1084/jem.190.7.1025 PMC2195646 10510092

[133] Thome M Schneider P Hofmann K Fickenscher H Meinl E Neipel F. Viral FLICE-Inhibitory Proteins (FLIPs) Prevent Apoptosis Induced by Death Receptors. Nature (1997) 386:517–21. doi: 10.1038/386517a0 9087414

[134] Micheau O Thome M Schneider P Holler N Tschopp J Nicholson DW. The Long Form of FLIP Is an Activator of Caspase-8 at the Fas Death-Inducing Signaling Complex. J Biol Chem (2002) 277:45162–71. doi: 10.1074/jbc.M206882200 12215447

[135] Yu JW Jeffrey PD Shi Y. Mechanism of Procaspase-8 Activation by C-FLIPL. Proc Natl Acad Sci (2009) 106:8169. doi: 10.1073/pnas.0812453106 19416807 PMC2688887

[136] Chang DW Xing Z Pan Y Algeciras-Schimnich A Barnhart BC Yaish-Ohad S. C-FLIPL is a Dual Function Regulator for Caspase-8 Activation and CD95-Mediated Apoptosis. EMBO J (2002) 21:3704–14. doi: 10.1093/emboj/cdf356 PMC125398 12110583

[137] Humphreys LM Fox JP Higgins CA Majkut J Sessler T McLaughlin K. A Revised Model of TRAIL-R2 DISC Assembly Explains How FLIP(L) can Inhibit or Promote Apoptosis. EMBO Rep (2020) 21:e49254. doi: 10.15252/embr.201949254 32009295 PMC7054686

[138] Higgins CA Fox J Roberts J Doherty D Perrior T Boffey R. Abstract 1342: Development and Preclinical Evaluation of Unique First-in-Class Small Molecule Inhibitors of the Anti-Apoptotic Protein FLIP. Cancer Res (2021) 81:1342–2. doi: 10.1158/1538-7445.AM2021-1342

[139] Safa AR Pollok KE. Targeting the Anti-Apoptotic Protein C-FLIP for Cancer Therapy. Cancers (Basel) (2011) 3:1639–71. doi: 10.3390/cancers3021639 PMC3281420 22348197

[140] El-Zawahry A McKillop J Voelkel-Johnson C. Doxorubicin Increases the Effectiveness of Apo2L/TRAIL for Tumor Growth Inhibition of Prostate Cancer Xenografts. BMC Cancer (2005) 5:2. doi: 10.1186/1471-2407-5-2 15638938 PMC546011

[141] Kim Y Suh N Sporn M Reed JC. An Inducible Pathway for Degradation of FLIP Protein Sensitizes Tumor Cells to TRAIL-Induced Apoptosis. J Biol Chem (2002) 277:22320–9. doi: 10.1074/jbc.M202458200 11940602

[142] Hyer ML Croxton R Krajewska M Krajewski S Kress CL Lu M. Synthetic Triterpenoids Cooperate With Tumor Necrosis Factor–Related Apoptosis-Inducing Ligand to Induce Apoptosis of Breast Cancer Cells. Cancer Res (2005) 65:4799–808. doi: 10.1158/0008-5472.CAN-04-3319 15930300

[143] Ashkenazi A Dixit VM. Apoptosis Control by Death and Decoy Receptors. Curr Opin Cell Biol (1999) 11:255–60. doi: 10.1016/S0955-0674(99)80034-9 10209153

[144] Mantovani A Locati M Vecchi A Sozzani S Allavena P. Decoy Receptors: A Strategy to Regulate Inflammatory Cytokines and Chemokines. Trends Immunol (2001) 22:328–36. doi: 10.1016/S1471-4906(01)01941-X 11377293

[145] Pitti RM Marsters SA Lawrence DA Roy M Kischkel FC Dowd P. Genomic Amplification of a Decoy Receptor for Fas Ligand in Lung and Colon Cancer. Nature (1998) 396:699–703. doi: 10.1038/25387 9872321

[146] Bai C Connolly B Metzker ML Hilliard CA Liu X Sandig V. Overexpression of M68/DcR3 in Human Gastrointestinal Tract Tumors Independent of Gene Amplification and its Location in a Four-Gene Cluster. Proc Natl Acad Sci (2000) 97:1230. doi: 10.1073/pnas.97.3.1230 10655513 PMC15578

[147] Degli-Esposti MA Dougall WC Smolak PJ Waugh JY Smith CA Goodwin RG. The Novel Receptor TRAIL-R4 Induces NF-κB and Protects Against TRAIL-Mediated Apoptosis, Yet Retains an Incomplete Death Domain. Immunity (1997) 7:813–20. doi: 10.1016/S1074-7613(00)80399-4 9430226

[148] Degli-Esposti MA Smolak PJ Walczak H Waugh J Huang C-P DuBose RF. Cloning and Characterization of TRAIL-R3, a Novel Member of the Emerging TRAIL Receptor Family. J Exp Med (1997) 186:1165–70. doi: 10.1084/jem.186.7.1165 PMC2199077 9314565

[149] Mérino D Lalaoui N Morizot A Schneider P Solary E Micheau O. Differential Inhibition of TRAIL-Mediated DR5-DISC Formation by Decoy Receptors 1 and 2. Mol Cell Biol (2006) 26:7046–55. doi: 10.1128/MCB.00520-06 PMC1592888 16980609

[150] Clancy L Mruk K Archer K Woelfel M Mongkolsapaya J Screaton G. Preligand Assembly Domain-Mediated Ligand-Independent Association Between TRAIL Receptor 4 (TR4) and TR2 Regulates TRAIL-Induced Apoptosis. Proc Natl Acad Sci U S A (2005) 102:18099. doi: 10.1073/pnas.0507329102 16319225 PMC1312398

[151] Ganten TM Sykora J Koschny R Batke E Aulmann S Mansmann U. Prognostic Significance of Tumour Necrosis Factor-Related Apoptosis-Inducing Ligand (TRAIL) Receptor Expression in Patients With Breast Cancer. J Mol Med (2009) 87:995. doi: 10.1007/s00109-009-0510-z 19680616

[152] Koksal IT Sanlioglu AD Karacay B Griffith TS Sanlioglu S. Tumor Necrosis Factor-Related Apoptosis Inducing Ligand-R4 Decoy Receptor Expression is Correlated With High Gleason Scores, Prostate-Specific Antigen Recurrence, and Decreased Survival in Patients With Prostate Carcinoma. Urol Oncol Semin Orig Investig (2008) 26:158–65. doi: 10.1016/j.urolonc.2007.01.022 18312935

[153] Chamuleau MED Ossenkoppele GJ van Rhenen A van Dreunen L Jirka SMG Zevenbergen A. High TRAIL-R3 Expression on Leukemic Blasts is Associated With Poor Outcome and Induces Apoptosis-Resistance Which can be Overcome by Targeting TRAIL-R2. Leuk Res (2011) 35:741–9. doi: 10.1016/j.leukres.2010.12.032 21281967

[154] Tarragona J Llecha N Santacana M Lopez S Gatius S Llobet D. DcR1 Expression in Endometrial Carcinomas. Virchows Arch (2010) 456:39–44. doi: 10.1007/s00428-009-0855-2 19936781

[155] Yu K-Y Kwon B Ni J Zhai Y Ebner R Kwon BS. A Newly Identified Member of Tumor Necrosis Factor Receptor Superfamily (TR6) Suppresses LIGHT-Mediated Apoptosis. J Biol Chem (1999) 274:13733–6. doi: 10.1074/jbc.274.20.13733 10318773

[156] Kriegl L Jung A Engel J Jackstadt R Gerbes AL Gallmeier E. Expression, Cellular Distribution, and Prognostic Relevance of TRAIL Receptors in Hepatocellular Carcinoma. Clin Cancer Res (2010) 16:5529. doi: 10.1158/1078-0432.CCR-09-3403 20889918

[157] Paschall AV Yang D Lu C Choi J-H Li X Liu F. H3K9 Trimethylation Silences Fas Expression To Confer Colon Carcinoma Immune Escape and 5-Fluorouracil Chemoresistance. J Immunol (2015) 195:1868. doi: 10.4049/jimmunol.1402243 26136424 PMC4530033

[158] Liu F Bardhan K Yang D Thangaraju M Ganapathy V Waller JL. NF-kB Directly Regulates Fas Transcription to Modulate Fas-Mediated Apoptosis and Tumor Suppression. J Biol Chem (2012) 287:25530–40. doi: 10.1074/jbc.M112.356279 PMC3408167 22669972

[159] Gazin C Wajapeyee N Gobeil S Virbasius C-M Green MR. An Elaborate Pathway Required for Ras-Mediated Epigenetic Silencing. Nature (2007) 449:1073–7. doi: 10.1038/nature06251 PMC2147719 17960246

[160] Peli J Schröter M Rudaz C Hahne M Meyer C Reichmann E. Oncogenic Ras Inhibits Fas Ligand-Mediated Apoptosis by Downregulating the Expression of Fas. EMBO J (1999) 18:1824–31. doi: 10.1093/emboj/18.7.1824 PMC1171268 10202146

[161] van Noesel MM van Bezouw S Salomons GS Voûte PA Pieters R Baylin SB. Tumor-Specific Down-Regulation of the Tumor Necrosis Factor-Related Apoptosis-Inducing Ligand Decoy Receptors DcR1 and DcR2 Is Associated With Dense Promoter Hypermethylation. Cancer Res (2002) 62:2157. 11929838

[162] Maecker HL Yun Z Maecker HT Giaccia AJ. Epigenetic Changes in Tumor Fas Levels Determine Immune Escape and Response to Therapy. Cancer Cell (2002) 2:139–48. doi: 10.1016/S1535-6108(02)00095-8 12204534

[163] Xu Y He B Li R Pan Y Gao T Deng Q. Association of the Polymorphisms in the Fas/FasL Promoter Regions With Cancer Susceptibility: A Systematic Review and Meta-Analysis of 52 Studies. PLoS One (2014) 9:e90090. doi: 10.1371/journal.pone.0090090 24598538 PMC3943814

[164] Jin Z McDonald ER III Dicker DT El-Deiry WS. Deficient Tumor Necrosis Factor-Related Apoptosis-Inducing Ligand (TRAIL) Death Receptor Transport to the Cell Surface in Human Colon Cancer Cells Selected for Resistance to TRAIL-Induced Apoptosis. J Biol Chem (2004) 279:35829–39. doi: 10.1074/jbc.M405538200 15155747

[165] Chinnaiyan AM Prasad U Shankar S Hamstra DA Shanaiah M Chenevert TL. Combined Effect of Tumor Necrosis Factor-Related Apoptosis-Inducing Ligand and Ionizing Radiation in Breast Cancer Therapy. Proc Natl Acad Sci U S A (2000) 97:1754–9. doi: 10.1073/pnas.030545097 PMC26508 10677530

[166] Marini P Schmid A Jendrossek V Faltin H Daniel PT Budach W. Irradiation Specifically Sensitises Solid Tumour Cell Lines to TRAIL Mediated Apoptosis. BMC Cancer (2005) 5:5. doi: 10.1186/1471-2407-5-5 15651986 PMC547906

[167] Chakraborty M Abrams SI Camphausen K Liu K Scott T Coleman CN. Irradiation of Tumor Cells Up-Regulates Fas and Enhances CTL Lytic Activity and CTL Adoptive Immunotherapy. J Immunol (2003) 170:6338–47. doi: 10.4049/JIMMUNOL.170.12.6338 12794167

[168] Garnett CT Palena C Chakraborty M Chakarborty M Tsang K-Y Schlom J. Sublethal Irradiation of Human Tumor Cells Modulates Phenotype Resulting in Enhanced Killing by Cytotoxic T Lymphocytes. Cancer Res (2004) 64:7985–94. doi: 10.1158/0008-5472.CAN-04-1525 15520206

[169] Ames E Canter RJ Grossenbacher SK Mac S Smith RC Monjazeb AM. Enhanced Targeting of Stem-Like Solid Tumor Cells With Radiation and Natural Killer Cells. Oncoimmunology (2015) 4:e1036212. doi: 10.1080/2162402X.2015.1036212 26405602 PMC4570100

[170] Hamasu T Inanami O Asanuma T Kuwabara M. Enhanced Induction of Apoptosis by Combined Treatment of Human Carcinoma Cells With X Rays and Death Receptor Agonists. J Radiat Res (2005) 46(1):103–10. 10.1269/jrr.46.103 15802865

[171] Amm HM Oliver PG Lee CH Li Y Buchsbaum DJ. Combined Modality Therapy With TRAIL or Agonistic Death Receptor Antibodies. Cancer Biol Ther (2011) 11:431–49. doi: 10.4161/cbt.11.5.14671 PMC3087899 21263219

[172] Lee SH Shin MS Kim HS Lee HK Park WS Kim SY. Somatic Mutations of TRAIL-Receptor 1 and TRAIL-Receptor 2 Genes in Non-Hodgkin’s Lymphoma. Oncogene (2001) 20:399–403. doi: 10.1038/sj.onc.1204103 11313970

[173] Shin MS Kim HS Lee SH Park WS Kim SY Park JY. Mutations of Tumor Necrosis Factor-Related Apoptosis-Inducing Ligand Receptor 1 (TRAIL-R1) and Receptor 2 (TRAIL-R2) Genes in Metastatic Breast Cancers. Cancer Res (2001) 61:4942–6. 11431320

[174] Park WS Lee JH Shin MS Park JY Kim HS Kim YS. Inactivating Mutations of KILLER/DR5 Gene in Gastric Cancers. Gastroenterology (2001) 121:1219–25. doi: 10.1053/gast.2001.28663 11677215

[175] Pai SI Wu GS Özören N Wu L Jen J Sidransky D. Rare Loss-Of-Function Mutation of a Death Receptor Gene in Head and Neck Cancer. Cancer Res (1998) 58:3513. 9721851

[176] Kearney CJ Vervoort SJ Hogg SJ Ramsbottom KM Freeman AJ Lalaoui N. Tumor Immune Evasion Arises Through Loss of TNF Sensitivity. Sci Immunol (2018) 3:eaar3451. doi: 10.1126/sciimmunol.aar3451 29776993

[177] Vredevoogd DW Kuilman T Ligtenberg MA Boshuizen J Stecker KE de Bruijn B. Augmenting Immunotherapy Impact by Lowering Tumor TNF Cytotoxicity Threshold. Cell (2019) 178:585–599.e15. doi: 10.1016/j.cell.2019.06.014 31303383

[178] Manguso RT Pope HW Zimmer MD Brown FD Yates KB Miller BC. *In Vivo* CRISPR Screening Identifies Ptpn2 as a Cancer Immunotherapy Target. Nature (2017) 547:413–8. doi: 10.1038/nature23270 PMC5924693 28723893

[179] Singh N Lee YG Shestova O Ravikumar P Hayer KE Hong SJ. Impaired Death Receptor Signaling in Leukemia Causes Antigen-Independent Resistance by Inducing CAR T-Cell Dysfunction. Cancer Discov (2020) 10:552–67. doi: 10.1158/2159-8290.CD-19-0813 PMC7416790 32001516

[180] Freeman AJ Vervoort SJ Michie J Ramsbottom KM Silke J Kearney CJ. HOIP Limits Anti-Tumor Immunity by Protecting Against Combined TNF and IFN-Gamma-Induced Apoptosis. EMBO Rep (2021) 22:e53391. doi: 10.15252/embr.202153391 34467615 PMC8567220

[181] Haas TL Emmerich CH Gerlach B Schmukle AC Cordier SM Rieser E. Recruitment of the Linear Ubiquitin Chain Assembly Complex Stabilizes the TNF-R1 Signaling Complex and Is Required for TNF-Mediated Gene Induction. Mol Cell (2009) 36:831–44. doi: 10.1016/j.molcel.2009.10.013 20005846

[182] Amir M Zhao E Fontana L Rosenberg H Tanaka K Gao G. Inhibition of Hepatocyte Autophagy Increases Tumor Necrosis Factor-Dependent Liver Injury by Promoting Caspase-8 Activation. Cell Death Differ (2013) 20:878–87. doi: 10.1038/cdd.2013.21 PMC3679456 23519075

[183] Hou W Han J Lu C Goldstein LA Rabinowich H. Autophagic Degradation of Active Caspase-8: A Crosstalk Mechanism Between Autophagy and Apoptosis. Autophagy (2010) 6:891–900. doi: 10.4161/auto.6.7.13038 20724831 PMC3039736

[184] Moore RJ Owens DM Stamp G Arnott C Burke F East N. Mice Deficient in Tumor Necrosis Factor-α are Resistant to Skin Carcinogenesis. Nat Med (1999) 5:828–31. doi: 10.1038/10552 10395330

[185] Orosz P Echtenacher B Falk W Rüschoff J Weber D Männel DN. Enhancement of Experimental Metastasis by Tumor Necrosis Factor. J Exp Med (1993) 177:1391–8. doi: 10.1084/jem.177.5.1391 PMC2191015 8478614

[186] Freeman AJ Kearney CJ Silke J Oliaro J. Unleashing TNF Cytotoxicity to Enhance Cancer Immunotherapy. Trends Immunol (2021) 42:1128–42. doi: 10.1016/j.it.2021.10.003 34750058

[187] Bai L Smith DC Wang S. Small-Molecule SMAC Mimetics as New Cancer Therapeutics. Pharmacol Ther (2014) 144:82–95. doi: 10.1016/j.pharmthera.2014.05.007 24841289 PMC4247261

[188] Dufva O Koski J Maliniemi P Ianevski A Klievink J Leitner J. Integrated Drug Profiling and CRISPR Screening Identify Essential Pathways for CAR T-Cell Cytotoxicity. Blood (2020) 135:597–609. doi: 10.1182/blood.2019002121 31830245 PMC7098811

[189] Kearney CJ Lalaoui N Freeman AJ Ramsbottom KM Silke J Oliaro J. PD-L1 and IAPs Co-Operate to Protect Tumors From Cytotoxic Lymphocyte-Derived TNF. Cell Death Differ (2017) 24:1705–16. doi: 10.1038/cdd.2017.94 PMC5596429 28665401

[190] Lin L Mathew TR Hidetaka S De Brabander JK Xiaodong W Harran PG. A Small Molecule Smac Mimic Potentiates TRAIL- and Tnfα-Mediated Cell Death. Science (80-) (2004) 305:1471–4. doi: 10.1126/science.1098231 15353805

[191] Olsson M Zhivotovsky B. Caspases and Cancer. Cell Death Differ (2011) 18:1441–9. doi: 10.1038/cdd.2011.30 PMC3178435 21455218

[192] Stupack DG. Caspase-8 as a Therapeutic Target in Cancer. Cancer Lett (2013) 332:133–40. doi: 10.1016/j.canlet.2010.07.022 PMC3049203 20817393

[193] Hopkins-Donaldson S Bodmer J-L Bourloud KB Brognara CB Tschopp J Gross N. Loss of Caspase-8 Expression in Highly Malignant Human Neuroblastoma Cells Correlates With Resistance to Tumor Necrosis Factor-Related Apoptosis-Inducing Ligand-Induced Apoptosis. Cancer Res (2000) 60:4315–9. 10969767

[194] Grotzer MA Eggert A Zuzak TJ Janss AJ Marwaha S Wiewrodt BR. Resistance to TRAIL-Induced Apoptosis in Primitive Neuroectodermal Brain Tumor Cells Correlates With a Loss of Caspase-8 Expression. Oncogene (2000) 19:4604–10. doi: 10.1038/sj.onc.1203816 11030149

[195] Deveraux QL Reed JC. IAP Family Proteins—Suppressors of Apoptosis. Genes Dev (1999) 13:239–52. doi: 10.1101/gad.13.3.239 9990849

[196] Sutton VR Wowk ME Cancilla M Trapani JA. Caspase Activation by Granzyme B Is Indirect, and Caspase Autoprocessing Requires the Release of Proapoptotic Mitochondrial Factors. Immunity (2003) 18:319–29. doi: 10.1016/S1074-7613(03)00050-5 12648450

[197] Goping IS Barry M Liston P Sawchuk T Constantinescu G Michalak KM. Granzyme B-Induced Apoptosis Requires Both Direct Caspase Activation and Relief of Caspase Inhibition. Immunity (2003) 18:355–65. doi: 10.1016/S1074-7613(03)00032-3 12648453

[198] Waterhouse NJ Sedelies KA Browne KA Wowk ME Newbold A Sutton VR. A Central Role for Bid in Granzyme B-Induced Apoptosis. J Biol Chem (2005) 280:4476–82. doi: 10.1074/jbc.M410985200 15574417

[199] Wang GQ Wieckowski E Goldstein LA Gastman BR Rabinovitz A Gambotto A. Resistance to Granzyme B-Mediated Cytochrome C Release in Bak-Deficient Cells. J Exp Med (2001) 194:1325–37. doi: 10.1084/jem.194.9.1325 PMC2195982 11696597

[200] Sinicrope FA Rego RL Foster NR Thibodeau SN Alberts SR Windschitl HE. Proapoptotic Bad and Bid Protein Expression Predict Survival in Stages II and III Colon Cancers. Clin Cancer Res (2008) 14:4128–33. doi: 10.1158/1078-0432.CCR-07-5160 PMC2948489 18593990

[201] Krajewska M Zapata JM Meinhold-Heerlein I Hedayat H Monks A Bettendorf H. Expression of Bcl-2 Family Member Bid in Normal and Malignant Tissues. Neoplasia (2002) 4:129–40. doi: 10.1038/sj.neo.7900222 PMC1550319 11896568

[202] Sutton VR Vaux DL Trapani JA. Bcl-2 Prevents Apoptosis Induced by Perforin and Granzyme B, But Not That Mediated by Whole Cytotoxic Lymphocytes. J Immunol (1997) 158:5783–90. 9190929

[203] Chiu VK Walsh CM Liu CC Reed JC Clark WR. Bcl-2 Blocks Degranulation But Not Fas-Based Cell-Mediated Cytotoxicity. J Immunol (1995) 154:2023. 7532659

[204] Sedelies KA Ciccone A Clarke CJP Oliaro J Sutton VR Scott FL. Blocking Granule-Mediated Death by Primary Human NK Cells Requires Both Protection of Mitochondria and Inhibition of Caspase Activity. Cell Death Differ (2008) 15:708–17. doi: 10.1038/sj.cdd.4402300 18202705

[205] Lickliter JD Cox J McCarron J Martinez NR Schmidt CW Lin H. Small-Molecule Bcl-2 Inhibitors Sensitise Tumour Cells to Immune-Mediated Destruction. Br J Cancer (2007) 96:600–8. doi: 10.1038/sj.bjc.6603599 PMC2360057 17311012

[206] Cingöz A Ozyerli-Goknar E Morova T Seker-Polat F Esai Selvan M Gümüş ZH. Generation of TRAIL-Resistant Cell Line Models Reveals Distinct Adaptive Mechanisms for Acquired Resistance and Re-Sensitization. Oncogene (2021) 40:3201–16. doi: 10.1038/s41388-021-01697-6 33767436

[207] Yip KW Reed JC. Bcl-2 Family Proteins and Cancer. Oncogene (2008) 27:6398–406. doi: 10.1038/onc.2008.307 18955968

[208] Kapoor I Bodo J Hill BT Hsi ED Almasan A. Targeting BCL-2 in B-Cell Malignancies and Overcoming Therapeutic Resistance. Cell Death Dis (2020) 11:941. doi: 10.1038/s41419-020-03144-y 33139702 PMC7608616

[209] Sánchez-Martínez D Azaceta G Muntasell A Aguiló N Núñez D Gálvez EM. Human NK Cells Activated by EBV+ Lymphoblastoid Cells Overcome Anti-Apoptotic Mechanisms of Drug Resistance in Haematological Cancer Cells. Oncoimmunology (2015) 4:e991613. doi: 10.4161/2162402X.2014.991613 25949911 PMC4404803

[210] Jaime-Sánchez P Catalán E Uranga-Murillo I Aguiló N Santiago L M Lanuza P. Antigen-Specific Primed Cytotoxic T Cells Eliminate Tumour Cells *In Vivo* and Prevent Tumour Development, Regardless of the Presence of Anti-Apoptotic Mutations Conferring Drug Resistance. Cell Death Differ (2018) 25:1536–48. doi: 10.1038/s41418-018-0112-9 PMC6143514 29743559

